# Supplementary material for: Genome-wide analysis of dental caries and periodontitis combining clinical and self-reported data
Source: Nat Commun. 2019 Jun 24;10:2773. doi: 10.1038/s41467-019-10630-1 (PMC6591304; doi:10.1038/s41467-019-10630-1)
Supplement: Supplementary file 2 — Supplementary Information [file 41467_2019_10630_MOESM2_ESM.pdf]

Supplementary Information for

Genome-wide analysis of dental caries and periodontitis combining clinical and self-reported data

D. Shungin *et al*

Table of Contents

Supplementary Figures
Supplementary Figure 1: Manhattan plot of transcript-level association statistics for combined DMFS/dentures meta-analysis
Supplementary Figure 2: Manhattan plot of transcript-level association statistics for combined periodontitis/loose teeth meta-analysis

Supplementary Tables
Supplementary Table 1: Estimated genetic correlation between dental disease traits within GLIDE
Supplementary Table 2: Estimated genetic correlation between dental disease traits within UKB
Supplementary Table 3: Risk loci with multiple conditionally-independent signals of association in DMFS/dentures combined analysis
Supplementary Table 4: Genes mapped to the lead single-variant association signal at *C5orf66* using the FUMA annotation tool.
Supplementary Table 5: Summary of results of PhenoScanner cross-trait comparison at lead DMFS/dentures associated loci
Supplementary Table 6: Association between HLA haplotypes and dentures in UK Biobank
Supplementary Table 7: Tests for heterogeneity in genetic effects on DMFS between participants with and without periodontitis.
Supplementary Table 8: Tests for heterogeneity in DMFS genetic effect estimates between HCHS/SOL and other studies in GLIDE
Supplementary Table 9: Estimated genetic correlations between DMFS/dentures and diseases or traits in the LD-hub catalogue
Supplementary Table 10: Estimated genetic correlations between periodontitis/loose teeth and traits or diseases in the LDHub catalogue.
Supplementary Table 11: Estimated causal effect of DMFS/dentures on metabolic traits and cardiovascular outcomes using alternative MR estimation tools
Supplementary Table 12: Estimated causal effect of metabolic traits on DMFS/dentures in GSMR primary analysis
Supplementary Table 13: Estimated causal effect of metabolic traits on DMFS/dentures using alternative MR estimation tools
Supplementary Table 14: Estimated casual effect of BMI and fasting glucose on DMFS/dentures in multivariable IVW MR
Supplementary Table 15: Estimated causal effect of metabolic traits on periodontitis/loose teeth in GSMR primary analysis
Supplementary Table 16: Estimated causal effect metabolic traits on periodontitis/loose teeth using alternative MR estimation tools.
Supplementary Table 17: Source data for Figure 1a
Supplementary Table 18: Source data for Figure 1b

Supplementary Notes
Supplementary Note 1: Acknowledgements
Supplementary Note 2: Standardized regression coefficients
Supplementary Note 3: Indicative effect sizes
Supplementary Note 4: Mendelian randomization sensitivity analyses

Supplementary References

Supplementary Figure 1: Manhattan plot of transcript-level association statistics for combined DMFS/dentures meta-analysis

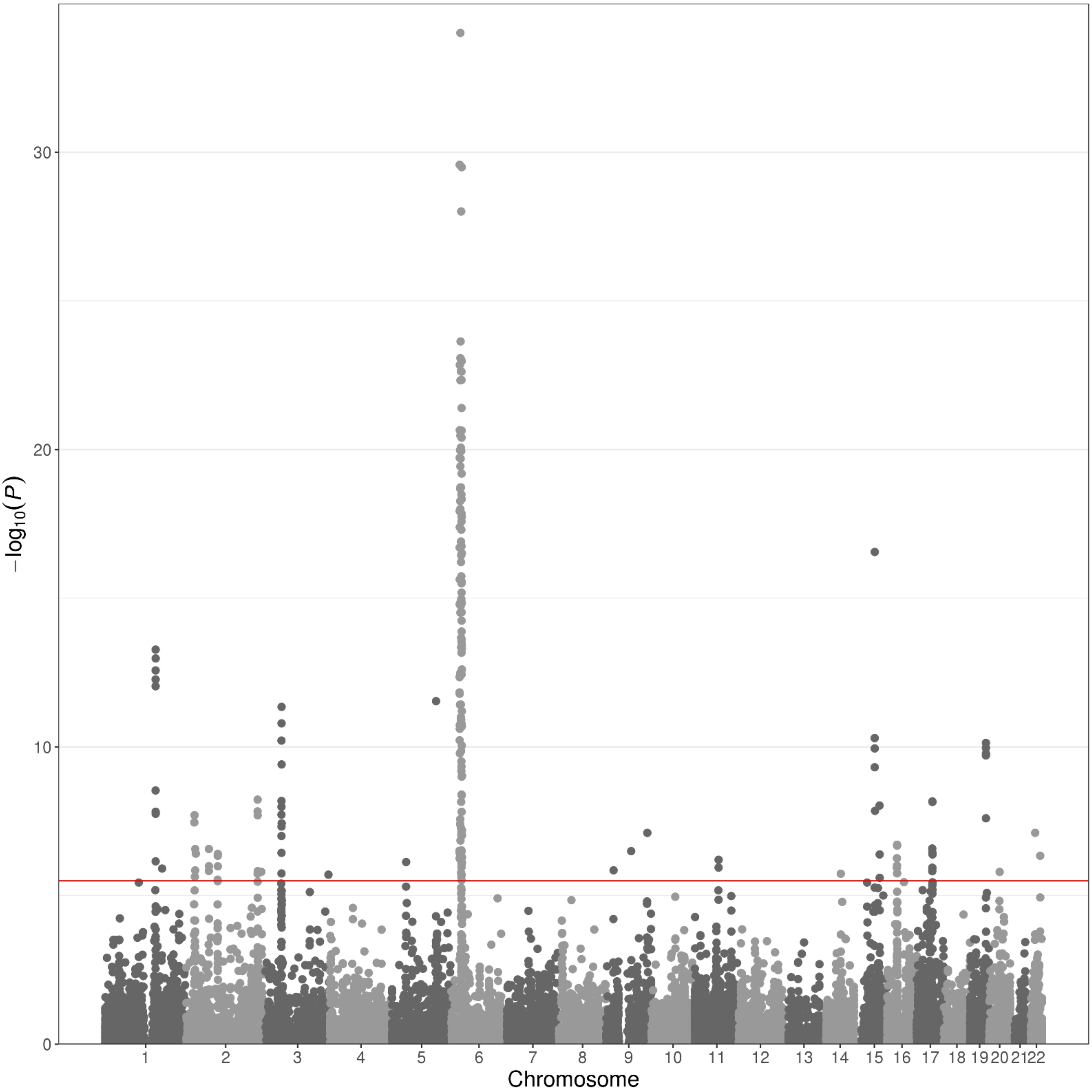

The red line indicates a Bonferroni-corrected multiple testing threshold at P=3.2x10^{-6}

Supplementary Figure 2: Manhattan plot of transcript-level association statistics for combined periodontitis/loose teeth meta-analysis

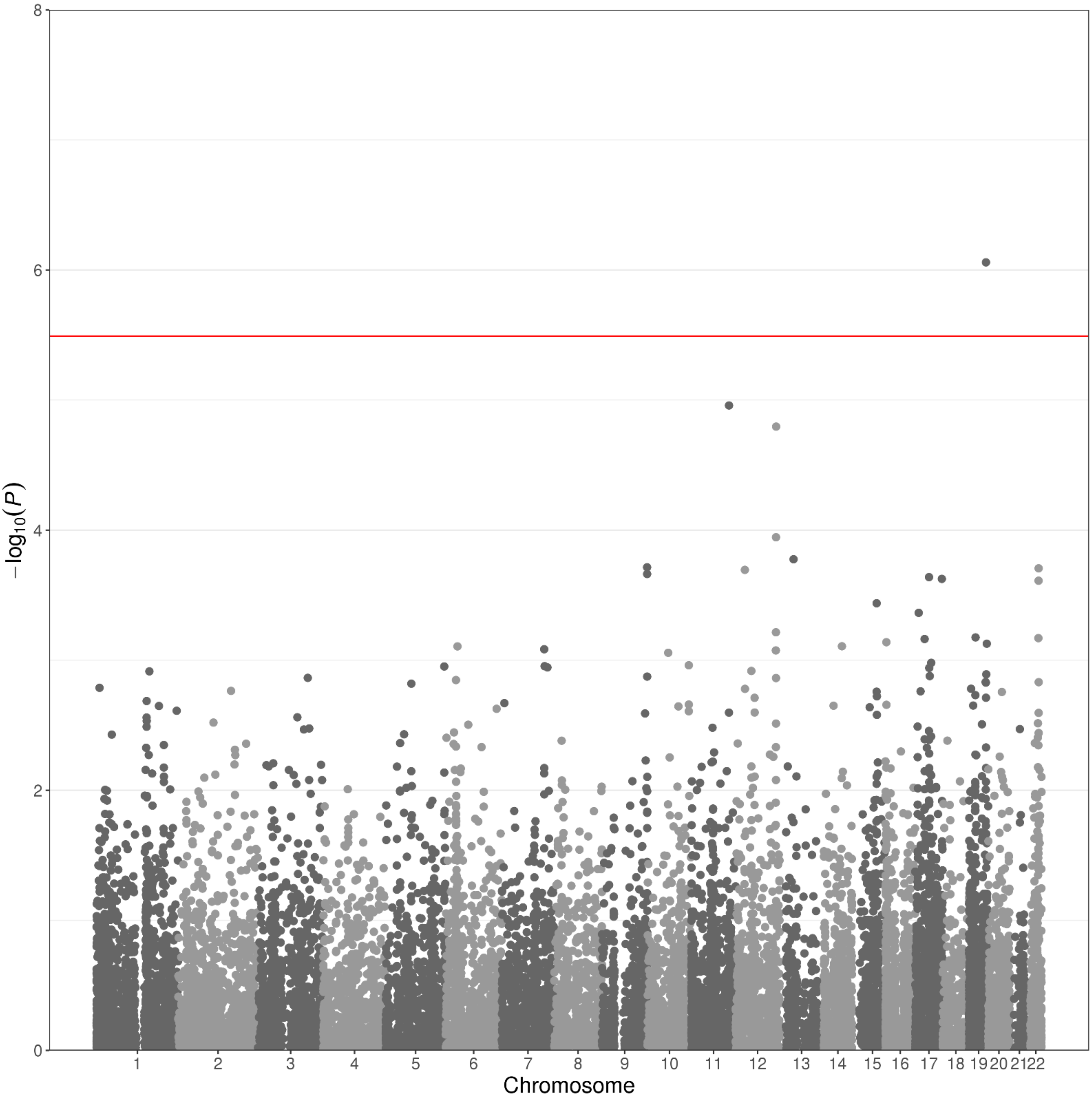

The red line indicates a Bonferroni-corrected multiple testing threshold at $P=3.2 \times 10^{-6}$

Supplementary Table 1: Estimated genetic correlation between dental disease traits within GLIDE

Trait 1	Trait 2	R_g	SE	p
DMFS	DFSS	1.14	0.13	2.0e-19
	Nteeth	-0.46	0.10	4.7e-6
	Periodontitis	-0.25	0.37	0.50
DFSS	Nteeth	-0.63	0.16	1.1e-4
	Periodontitis	0.60	0.60	0.32
Nteeth	Periodontitis	0.21	0.37	0.58

Supplementary Table 2: Estimated genetic correlation between dental disease traits within UKB

Trait 1	Trait 2	R_g	SE	p
Bleeding gums	Dentures	0.0009	0.035	0.98
	Loose teeth	0.37	0.055	2.8e-11
	Painful gums	0.50	0.069	2.0e-13
	Toothache	0.38	0.072	1.6e-7
	Ulcers	0.20	0.042	1.3e-6
Dentures	Loose teeth	0.46	0.043	9.6e-27
	Painful gums	0.14	0.054	7.3e-3
	Toothache	0.22	0.059	2.4e-4
	Ulcers	-0.082	0.036	0.0024
Loose teeth	Painful gums	0.46	0.088	1.3e-7
	Toothache	0.36	0.089	6.5e-5
	Ulcers	0.071	0.052	0.17
Painful gums	Toothache	0.87	0.11	3.2e-15
	Ulcers	0.59	0.066	5.9e-19
Toothache	Ulcers	0.43	0.065	3.4e-11

Supplementary Table 3: Risk loci with multiple conditionally-independent signals of association in DMFS/dentures combined analysis

Locus	Lead tag variant			Other variant		
	rsid	Unconditional P value	Conditional P value	rsid	Unconditional P value	Conditional P value
FAM150B	rs62106258	8.60E-12	3.40E-12	rs13028737	2.18E-8	8.53E-09
ALK	rs80270335	2.10E-09	3.50E-11	rs4128318	4.93E-6	4.85E-08
CA12	rs72748935	1.3E-26	2.6E-25	rs7180729	8.00E-10	1.91E-08

Supplementary Table 4. Genes mapped to the lead single-variant association signal at *C5orf66* using the FUMA annotation tool.

Gene	Chr	Start	End	Strand	posSNPs	eqtlSNPs	ciMap
GDF9	5	132196873	132202576	-1	0	0	Yes
UQCRQ	5	132202252	132203723	1	0	0	Yes
LEAP2	5	132208014	132210738	1	0	0	Yes
SKP1	5	133484633	133512729	-1	0	0	Yes
PPP2CA	5	133530025	133561833	-1	0	0	Yes
CDKL3	5	133541305	133706738	-1	0	0	Yes
UBE2B	5	133706870	133727683	1	0	0	Yes
CDKN2AIPNL	5	133737778	133747589	-1	0	0	Yes
JADE2	5	133860003	133918918	1	0	0	Yes
SAR1B	5	133936834	133984961	-1	2	3	No
SEC24A	5	133984479	134063513	1	1	1	No
CAMLG	5	134074191	134087847	1	1	25	No
DDX46	5	134094469	134190823	1	0	0	Yes
C5orf24	5	134181370	134195427	1	0	0	Yes
TXNDC15	5	134209493	134237215	1	0	46	No
PCBD2	5	134240596	134343649	1	38	0	Yes
CATSPER3	5	134303596	134347392	1	47	0	Yes
PITX1	5	134363425	134370503	-1	27	68	Yes
C5orf66	5	134368970	134691744	1	178	0	Yes
H2AFY	5	134669590	134735604	-1	0	0	Yes
C5orf20	5	134779905	134783038	-1	0	0	Yes
TIFAB	5	134779908	134788089	-1	0	0	Yes
SLC25A48	5	135170338	135224326	1	0	1	No
TGFBI	5	135364584	135399507	1	0	3	No
SMAD5	5	135468534	135524435	1	0	2	No

Start and end positions are given with reference to hg19. PosSNPs refers to the number of DMFS/dentures associated single variants mapping to a gene based on position alone. eqtlSNPs refers to the number of DMFS/dentures associates single variants which are reported to be eQTLs for the gene. ciMap indicates whether the gene has a reported chromatin interaction with a DMFS/dentures associated single variant in reference data.

Supplementary Table 5: Summary of results of PhenoScanner cross-trait comparison at lead DMFS/dentures associated variants

Target RSID	Reported association with non-oral health trait (p<5e-8)	Details
rs72694438	No	
rs4971099	Yes	Magnesium, urate, adiposity traits
rs2046850	Yes	Wheeze or whistling
rs3820640	No	
rs62106258	Yes	Adiposity traits and basal metabolic rate
rs11676272	Yes	Adiposity traits, height and FEV
rs80270335	No	
rs5831974	No	
rs2652452	Yes	Physical activity
rs263771	Yes	Physical activity
rs121908120	Yes	Hair loss
rs9831002	No	
rs7429279	Yes	Adiposity traits
rs61790808	Yes	Height, adiposity and personality traits
rs55769264	Yes	Educational attainment
rs1482698	No	
rs1352724	No	
rs1122171	Yes	Height
rs9366651	Yes	Height, adiposity traits, educational attainment
rs898797	Yes	Bone mineral density, adiposity traits, height and red blood cell traits
rs10811723	No	
rs7852129	No	
rs10987008	Yes	Subjective overall health, height
rs7918807	No	
rs149467613	Yes	Haematinic traits
rs10772314	No	
rs72748935	No	
rs6495046	Yes	Pulse rate, adiposity traits
rs10851907	Yes	Smoking traits and smoking-related diseases
rs2072693	No	
rs8054556	Yes	Basal metabolic rate and adiposity traits
rs1108343	Yes	Bone mineral density
rs10048146	Yes	Bone mineral density, height
rs3865314	Yes	Height and adiposity traits
rs9905793	No	
rs34559440	Yes	Adiposity traits
rs7217268	No	
rs57067187	Yes	Height, physical activity, FEV and educational attainment
rs28822480	Yes	Adiposity traits and basal metabolic rate
rs2238651	No	
rs11672900	Yes	Renal, urinary and haematinic traits
rs4816017	No	
rs140357883	No	
rs1569414	Yes	Height, facial hair and hair loss, adiposity traits
rs5922945	No	

Supplementary Table 6: Association between HLA haplotypes and dentures in UK Biobank

Haplotype	Beta	SE	Z	p	Odds Ratio (95% CI)	Haplotype frequency
**DRB3_101**	0.058	0.009	6.39	1.63E-10	1.06 (1.04, 1.08)	0.17
**DRB1_301**	0.067	0.009	7.10	1.24E-12	1.07 (1.05, 1.09)	0.15
**DRB1_101**	-0.059	0.012	-4.91	9.01E-07	0.94 (0.92, 0.97)	0.09
**DQB1_501**	-0.047	0.011	-4.45	8.49E-06	0.95 (0.93, 0.97)	0.12
**DQB1_201**	0.068	0.009	7.15	8.87E-13	1.07 (1.05, 1.09)	0.15
**DQA1_501**	0.037	0.008	4.50	6.87E-06	1.04 (1.02, 1.05)	0.23
**C_701**	0.046	0.009	5.17	2.37E-07	1.05 (1.03, 1.07)	0.18
**B_801**	0.061	0.010	6.38	1.72E-10	1.06 (1.04, 1.08)	0.14
**B_1501**	-0.054	0.014	-3.77	1.60E-04	0.95 (0.92, 0.97)	0.06
**A_101**	0.058	0.009	6.80	1.04E-11	1.06 (1.04, 1.08)	0.19

All models incorporated adjustment for age, sex, genotyping array and 40 genetic principal components.

Supplementary Table 7: Tests for heterogeneity in genetic effects on DMFS between participants with and without periodontitis.

RSID	Chr:Pos (b 37)	A1	A2	Periodontal controls		Periodontal cases		P_het	P_het_FDR*
				Beta	SE	Beta	SE		
rs72694438	1:104364878	a	g	0.004	0.018	0.045	0.018	0.11	0.97
rs4971099	1:155155608	a	g	-0.043	0.013	-0.014	0.014	0.12	0.97
rs2046850	1:210304319	t	c	-0.013	0.017	-0.008	0.017	0.83	0.97
rs3820640	1:226868918	t	c	-0.011	0.021	0.001	0.020	0.67	0.97
rs62106258	2:417167	t	c	0.069	0.039	0.079	0.039	0.86	0.97
rs11676272	2:25141538	a	g	-0.015	0.013	-0.006	0.014	0.61	0.97
rs80270335	2:29616655	t	c	0.096	0.026	0.024	0.026	0.05	0.92
rs2652452	2:155670203	a	c	-0.006	0.013	0.000	0.013	0.74	0.97
rs263771	2:185921692	a	c	-0.003	0.017	0.001	0.017	0.88	0.97
rs121908120	2:219755011	a	t	-0.062	0.061	-0.153	0.060	0.28	0.97
rs9831002	3:18852697	t	g	-0.014	0.014	0.001	0.014	0.44	0.97
rs7429279	3:25118637	a	c	0.001	0.014	-0.003	0.014	0.85	0.97
rs61790808	3:136443008	a	g	-0.039	0.017	-0.032	0.017	0.76	0.97
rs185566659	3:193394725	a	g	-0.021	0.047	-0.033	0.044	0.85	0.97
rs55769264	5:26928047	a	g	-0.005	0.015	0.004	0.015	0.68	0.97
rs1482698	5:44539453	c	g	0.040	0.014	0.020	0.014	0.32	0.97
rs1352724	5:107083487	a	c	0.015	0.019	0.007	0.018	0.77	0.97
rs1122171	5:134509987	t	c	0.065	0.013	0.043	0.013	0.23	0.97
rs9366651	6:26336696	t	g	-0.004	0.014	-0.014	0.013	0.58	0.97
rs898797	8:9229689	t	c	0.029	0.014	-0.005	0.014	0.09	0.97
rs10811723	9:22542285	a	g	0.008	0.014	-0.028	0.014	0.06	0.92
rs7852129	9:79346204	a	c	0.000	0.042	-0.044	0.047	0.48	0.97
rs10987008	9:128661600	a	t	0.004	0.014	0.020	0.014	0.41	0.97
rs7918807	10:10020194	t	c	0.031	0.013	0.018	0.013	0.50	0.97
rs149467613	11:72943483	a	g	-0.045	0.044	-0.061	0.044	0.80	0.97
rs10772314	12:10704350	a	t	-0.021	0.013	-0.016	0.013	0.80	0.97
rs72748935	15:63639416	t	c	-0.047	0.014	-0.044	0.014	0.91	0.97
rs6495046	15:73353175	c	g	-0.009	0.014	-0.004	0.014	0.82	0.97
rs10851907	15:78915864	a	g	0.004	0.014	0.023	0.014	0.33	0.97
rs2072693	15:90014945	t	g	0.043	0.014	0.004	0.014	0.04	0.92
rs8054556	16:29958216	a	g	0.027	0.014	0.019	0.014	0.66	0.97
rs1108343	16:51211595	t	c	0.001	0.015	-0.007	0.015	0.71	0.97
rs10048146	16:86710660	a	g	-0.032	0.018	-0.013	0.018	0.45	0.97
rs3865314	17:45669524	a	c	0.013	0.014	0.003	0.014	0.61	0.97
rs9905793	17:46635649	a	g	0.023	0.020	0.026	0.020	0.89	0.97
rs34559440	17:68399112	t	c	0.004	0.014	0.008	0.014	0.86	0.97
rs7217268	17:70338127	a	g	0.020	0.015	0.018	0.014	0.95	0.97
rs57067187	17:79361332	t	c	0.001	0.016	0.003	0.016	0.95	0.97
rs28822480	18:57924823	a	g	0.055	0.016	0.030	0.016	0.25	0.97
rs2238651	19:18718846	t	c	0.026	0.017	0.043	0.017	0.48	0.97
rs11672900	19:49220323	a	g	-0.017	0.014	-0.018	0.014	0.95	0.97
rs4816017	20:7654373	a	g	-0.032	0.014	-0.011	0.014	0.30	0.97
rs1569414	22:45727565	t	g	-0.017	0.014	-0.035	0.014	0.37	0.97

* P values after a Benjamini-Hochberg correction

Supplementary Table 8: Tests for heterogeneity in DMFS genetic effect estimates between HCHS/SOL and other studies in GLIDE

RSID	Chr:Pos (b 37)	A1	A2	GLIDE excluding HCHS/SOL		HCHS/SOL		P_het	P_het_FDR*
				Beta	SE	Beta	SE		
rs72694438	1:104364878	a	g	0.024	0.015	0.032	0.018	0.74	0.95
rs4971099	1:155155608	a	g	-0.030	0.012	-0.017	0.013	0.46	0.93
rs2046850	1:210304319	t	c	-0.002	0.014	-0.040	0.017	0.09	0.89
rs3820640	1:226868918	t	c	0.012	0.015	-0.013	0.024	0.38	0.89
rs62106258	2:417167	t	c	0.014	0.037	0.102	0.037	0.09	0.89
rs11676272	2:25141538	a	g	-0.016	0.012	-0.026	0.013	0.58	0.93
rs80270335	2:29616655	t	c	0.047	0.020	0.043	0.032	0.92	0.95
rs5831974	2:69704336:ID	d	i	-0.023	0.012	0.000	0.014	0.21	0.89
rs2652452	2:155670203	a	c	0.004	0.012	-0.016	0.013	0.27	0.89
rs263771	2:185921692	a	c	0.020	0.014	0.013	0.018	0.77	0.95
rs121908120	2:219755011	a	t	-0.125	0.050	-0.104	0.061	0.80	0.95
rs9831002	3:18852697	t	g	-0.015	0.012	-0.008	0.013	0.70	0.93
rs7429279	3:25118637	a	c	0.000	0.012	0.010	0.014	0.58	0.93
3:50135699:ID	3:50135699:ID	d	i	-0.007	0.012	-0.016	0.013	0.60	0.93
rs61790808	3:136443008	a	g	-0.012	0.015	-0.040	0.017	0.20	0.89
rs185566659	3:193394725	a	g	0.006	0.035	0.063	0.054	0.37	0.89
rs55769264	5:26928047	a	g	0.015	0.012	0.015	0.016	0.98	0.98
rs1482698	5:44539453	c	g	0.014	0.012	0.033	0.014	0.31	0.89
rs1352724	5:107083487	a	c	-0.019	0.015	0.012	0.020	0.21	0.89
rs1122171	5:134509987	t	c	0.074	0.012	0.051	0.013	0.18	0.89
rs9366651	6:26336696	t	g	-0.030	0.012	-0.025	0.013	0.78	0.95
rs898797	8:9229689	t	c	-0.005	0.012	0.014	0.014	0.30	0.89
rs10811723	9:22542285	a	g	-0.003	0.013	-0.012	0.013	0.63	0.93
rs7852129	9:79346204	a	c	-0.116	0.058	0.003	0.035	0.08	0.89
rs10987008	9:128661600	a	t	0.024	0.012	0.004	0.013	0.28	0.89
rs7918807	10:10020194	t	c	0.021	0.012	0.016	0.013	0.78	0.95
rs149467613	11:72943483	a	g	-0.071	0.037	-0.122	0.046	0.38	0.89
rs10772314	12:10704350	a	t	-0.014	0.012	-0.005	0.013	0.61	0.93
rs72748935	15:63639416	t	c	-0.070	0.012	-0.026	0.014	0.02	0.89
rs6495046	15:73353175	c	g	-0.037	0.012	-0.007	0.014	0.11	0.89
rs10851907	15:78915864	a	g	0.017	0.012	-0.004	0.014	0.26	0.89
rs2072693	15:90014945	t	g	0.009	0.012	0.037	0.013	0.13	0.89
rs8054556	16:29958216	a	g	0.014	0.012	0.034	0.014	0.28	0.89
rs1108343	16:51211595	t	c	0.006	0.013	0.008	0.014	0.92	0.95
rs10048146	16:86710660	a	g	-0.030	0.015	-0.013	0.020	0.48	0.93
rs3865314	17:45669524	a	c	-0.003	0.012	0.013	0.013	0.35	0.89
rs9905793	17:46635649	a	g	0.018	0.021	0.031	0.017	0.63	0.93
rs34559440	17:68399112	t	c	-0.007	0.012	0.007	0.014	0.44	0.93
rs7217268	17:70338127	a	g	0.008	0.013	0.012	0.014	0.82	0.95
rs57067187	17:79361332	t	c	0.010	0.014	0.019	0.016	0.66	0.93
rs28822480	18:57924823	a	g	0.060	0.013	0.016	0.017	0.04	0.89
rs2238651	19:18718846	t	c	0.022	0.014	0.023	0.019	0.96	0.98
rs11672900	19:49220323	a	g	0.002	0.012	-0.025	0.014	0.14	0.89
rs4816017	20:7654373	a	g	-0.015	0.013	-0.028	0.014	0.48	0.93
rs140357883	22:30292811:ID	d	i	0.029	0.017	0.019	0.020	0.70	0.93
rs1569414	22:45727565	t	g	-0.028	0.013	-0.031	0.013	0.89	0.95
rs5922945	23:83523015	t	c	-0.020	0.018	-0.023	0.011	0.86	0.95

* P values after a Benjamini-Hochberg correction

Supplementary Table 9: Estimated genetic correlations between DMFS/dentures and diseases or traits in the LD-hub catalogue

Trait	PMID	R_g	SE	P
Years of schooling 2016	27225129	-0.5236	0.0192	1.77E-163
Age of first birth	27798627	-0.5026	0.0303	5.61E-62
Years of schooling (proxy cognitive performance)	25201988	-0.5469	0.035	3.80E-55
Years of schooling 2013	23722424	-0.5492	0.0364	2.09E-51
College completion	23722424	-0.5249	0.0382	6.49E-43
Intelligence	28530673	-0.3281	0.0301	1.41E-27
Number of children ever born	27798627	0.3547	0.0347	1.59E-24
Ever vs never smoked	20418890	0.3777	0.0418	1.79E-19
Waist circumference	25673412	0.2321	0.026	3.93E-19
Waist-to-hip ratio	25673412	0.2501	0.0283	1.10E-18
Obesity class 1	23563607	0.2457	0.0287	1.10E-17
Overweight	23563607	0.2399	0.03	1.32E-15
Body mass index	20935630	0.2118	0.0269	3.24E-15
Former vs Current smoker	20418890	-0.5195	0.0687	3.92E-14
Fathers age at death	27015805	-0.4665	0.0635	2.01E-13
Lung cancer	27488534	0.3573	0.05	8.69E-13
Mothers age at death	27015805	-0.4926	0.0696	1.50E-12
Body fat	26833246	0.2804	0.04	2.28E-12
Obesity class 2	23563607	0.2549	0.0377	1.41E-11
Coronary artery disease	26343387	0.1939	0.0305	2.12E-10
Age at Menopause	26414677	-0.2145	0.0344	4.71E-10
Parents age at death	27015805	-0.4726	0.0773	9.55E-10
Childhood IQ	23358156	-0.3688	0.0606	1.16E-09
Hip circumference	25673412	0.1531	0.0254	1.66E-09
Lung cancer (all)	24880342	0.316	0.0541	5.08E-09
Extreme bmi	23563607	0.2444	0.043	1.28E-08
Squamous cell lung cancer	27488534	0.4583	0.0817	2.02E-08
Cigarettes smoked per day	20418890	0.3804	0.0706	7.11E-08
HDL cholesterol	20686565	-0.1854	0.0355	1.83E-07
Childhood obesity	22484627	0.2039	0.0429	2.02E-06
22:6 docosahexaenoic acid	27005778	-0.3402	0.0718	2.18E-06
Depressive symptoms	27089181	0.2163	0.0471	4.38E-06
Anorexia Nervosa	24514567	-0.146	0.0322	5.97E-06
Rheumatoid Arthritis	24390342	0.1751	0.0391	7.54E-06
Fasting glucose main effect	22581228	0.169	0.0381	9.16E-06
Bipolar disorder	21926972	-0.1584	0.036	1.10E-05
Obesity class 3	23563607	0.2058	0.0488	2.44E-05
Insomnia	28604731	0.197	0.0469	2.72E-05
Lung cancer (squamous cell)	24880342	0.431	0.1037	3.26E-05
Leptin_not_adjBMI	26833098	0.2047	0.0556	0.0002
HbA1C	20858683	0.2014	0.0549	0.0002
Attention deficit hyperactivity disorder (GC)	27663945	0.3914	0.1045	0.0002
Attention deficit hyperactivity disorder (No GC)	27663945	0.3913	0.1047	0.0002

Supplementary Table 10: Estimated genetic correlations between periodontitis/loose teeth and traits or diseases in the LDHub catalogue.

Trait	PMID	R_g	SE	P
Years of schooling 2016	27225129	-0.3718	0.0394	4.00E-21
Ever vs never smoked	20418890	0.6429	0.0708	1.12E-19
Age of first birth	27798627	-0.4435	0.0543	3.06E-16
Waist-to-hip ratio	25673412	0.3246	0.0448	4.42E-13
College completion	23722424	-0.35	0.0616	1.35E-08
Years of schooling 2013	23722424	-0.3659	0.0668	4.30E-08
Years of schooling (proxy cognitive performance)	25201988	-0.3409	0.0623	4.45E-08
Waist circumference	25673412	0.266	0.0496	8.40E-08
Body fat	26833246	0.3323	0.0629	1.27E-07
Overweight	23563607	0.2782	0.0541	2.77E-07
Depressive symptoms	27089181	0.3386	0.0659	2.82E-07
Former vs Current smoker	20418890	-0.5551	0.109	3.52E-07
Lung cancer	27488534	0.4318	0.0855	4.47E-07
Obesity class 1	23563607	0.2486	0.0504	7.95E-07
Intelligence	28530673	-0.2482	0.051	1.11E-06
Number of children ever born	27798627	0.2885	0.0615	2.69E-06
Body mass index	20935630	0.2474	0.0538	4.23E-06
Cigarettes smoked per day	20418890	0.4948	0.1087	5.29E-06
Lung cancer (all)	24880342	0.4343	0.0965	6.80E-06
Fathers age at death	27015805	-0.4535	0.1021	8.96E-06
Insomnia	28604731	0.341	0.0788	1.52E-05
Obesity class 2	23563607	0.2861	0.0664	1.66E-05
Mothers age at death	27015805	-0.4892	0.1145	1.94E-05
Extreme waist-to-hip ratio	23563607	0.4211	0.0998	2.47E-05
Schizophrenia	25056061	0.1689	0.041	3.71E-05
Extreme bmi	23563607	0.2905	0.0729	6.66E-05
Squamous cell lung cancer	27488534	0.5594	0.1405	6.86E-05
Childhood obesity	22484627	0.2955	0.0761	0.0001
Parents age at death	27015805	-0.4711	0.1228	0.0001
Subjective well being	27089181	-0.2561	0.0689	0.0002
Lung cancer (squamous cell)	24880342	0.6428	0.172	0.0002

Supplementary Table 11: Estimated causal effect of DMFS/dentures on metabolic traits and cardiovascular outcomes using alternative MR estimation tools

Trait	GSMR Beta (SE)	IVW Beta (SE)	MR Egger Beta (SE)	Model averaging Beta	95% CI for model averaging	99% CI for model averaging
BMI	-0.006 (0.019)	0.094 (0.070)	-0.38 (0.23)	-0.06	-0.10, -0.01	-0.12, 0.01
WHR *adj* BMI	0.11 (0.036)	0.094 (0.047)	0.29 (0.17)	0.12	0.01, 0.22	-0.03, 0.27
Type 2 Diabetes	0.25 (0.10)	0.28 (0.15)	0.48 (0.49)	0.16	-0.24, 0.57	-0.35, 0.88
Fasting glucose	0.004 (0.034)	0.006 (0.035)	0.084 (0.12)	-0.06	-0.15, 0.05	-0.18, 0.08
HDL-c	-0.030 (0.041)	-0.12 (0.070)	0.31 (0.25)	-0.14	-0.25, -0.004	-0.31, 0.04
LDL-c	0.023 (0.043)	0.067 (0.064)	-0.053 (0.24)	0.084	-0.08, 0.29	-0.12, 0.43
Triglycerides	0.11 (0.038)	0.11 (0.049)	-0.24 (0.17)	0.25	0.12, 0.42	-0.11, 0.47
Coronary artery disease	0.13 (0.079)	0.17 (0.11)	-0.51 (0.34)	0.20	-0.40, 0.90	-0.51, 1.1
All Stroke	0.16 (0.077)	0.18 (0.081)	0.11 (0.26)	0.046	-0.20, 0.57	-0.29, 0.69

Supplementary Table 12: Estimated causal effect of metabolic traits on DMFS/dentures in GSMR primary analysis

Trait	Untransformed Beta (SE)	Transformed Beta (95% CI)*	Units for exposure	NSNP	P
BMI	0.13 (0.007)	0.78 (0.70, 0.86)	KgM^{-2}	804	$6.0x10^{-75}$
Waist Hip Ratio adjusted for BMI	-0.017 (0.016)	-0.46 (-1.3, 0.4)	SD INT	47	0.30
Fasting glucose	0.040 (0.016)	1.1 (0.2, 2.0)	mM	23	0.015
HDL-c	-0.0001 (0.006)	-0.004 (-0.3, 0.3)	SD INT	159	0.98
LDL-c	-0.003 (0.005)	-0.08 (-0.4, 0.2)	SD INT	132	0.58
Triglycerides	-0.007 (0.008)	-0.19 (-0.6, 0.2)	SD INT	95	0.37

*All transformed effects are expressed in tooth surfaces per unit of exposure.

Supplementary Table 13: Estimated causal effect of metabolic traits on DMFS/dentures using alternative MR estimation tools

Trait	GSMR Beta (SE)	IVW Beta (SE)	MR Egger Beta (SE)	Model averaging Beta	95% CI for model averaging	99% CI for model averaging
BMI	0.13 (0.007)	0.14 (0.01)	0.14 (0.03)	0.22	0.19,0.24	0.18, 0.25
WHR *adj* BMI	-0.017 (0.016)	-0.012 (0.018)	-0.042 (0.072)	-0.026	-0.08, 0.03	-0.11, 0.05
Fasting glucose	0.040 (0.016)	0.047 (0.023)	0.091 (0.051)	0.063	0.027, 0.11	0.009, 0.12
HDL-c	-0.0001 (0.006)	-0.05 (0.07)	0.006 (0.012)	-0.0053	-0.016, 0.008	-0.020, 0.012
LDL-c	-0.003 (0.005)	-0.002 (0.008)	0.012 (0.013)	0.0006	-0.010, 0.011	-0.014, 0.015
Triglycerides	-0.007 (0.008)	-0.027 (0.011)	-0.024 (0.018)	-0.015	-0.031, 0.006	-0.036, 0.012

Supplementary Table 14: Estimated casual effect of BMI and fasting glucose on DMFS/dentures in multivariable IVW MR

Trait	MV IVW Beta (SE)	P
BMI	0.14 (0.01)	$3.5x10^{-38}$
Fasting glucose	0.047 (0.023)	0.006

Supplementary Table 15: Estimated causal effect of metabolic traits on periodontitis/loose teeth in GSMR primary analysis

Trait	Untransformed Beta (SE)	OR (95% CI)*	Units for exposure	NSNP	P
BMI	0.09 (0.01)	1.05 (1.04, 1.06)	KgM^{-2}	854	$3.0x10^{-18}$
Waist Hip Ratio adjusted for BMI	0.015 (0.025)	1.04 (0.92, 1.16)	SD INT	48	0.55
Fasting glucose	0.028 (0.026)	1.06 (0.95, 1.20)	mM	23	0.28
HDL-c	0.015 (0.009)	1.04 (1.00, 1.08)	SD INT	169	0.083
LDL-c	0.006 (0.008)	1.01 (0.97, 1.05)	SD INT	145	0.51
Triglycerides	0.006 (0.011)	1.01 (0.96, 1.07)	SD INT	104	0.58

*All odds ratios are expressed per unit of exposure.

Supplementary Table 16: Estimated causal effect metabolic traits on periodontitis/loose teeth using alternative MR estimation tools.

Trait	GSMR Beta (SE)	IVW Beta (SE)	MR Egger Beta (SE)	Model Averaging Beta	95% CI for model averaging	99% CI for model averaging
BMI	0.09 (0.01)	0.12 (0.01)	0.11 (0.036)	0.14	0.11, 0.18	0.09, 0.19
WHR *adj* BMI	0.015 (0.025)	0.014 (0.029)	0.021 (0.11)	0.005	-0.05, 0.06	-0.08, 0.09
Fasting glucose	0.028 (0.026)	0.027 (0.035)	0.071 (0.078)	0.11	0.055, 0.18	-0.10, 0.21
HDL-c	0.015 (0.009)	0.016 (0.008)	0.027 (0.013)	0.024	0.002, 0.045	-0.006, 0.05
LDL-c	0.006 (0.008)	0.004 (0.008)	0.004 (0.013)	0.009	-0.008, 0.03	-0.015, 0.033
Triglycerides	0.006 (0.011)	0.009 (0.011)	0.016 (0.019)	0.012	-0.018, 0.043	-0.027, 0.057

Supplementary Table 17. Source data for Figure 1a (Estimated heritability).

Resource	Trait	h^2_{LDSR}	SE
GLIDE	DMFS	0.090	0.018
	DFSS	0.057	0.017
	Nteeth	0.13	0.019
	Periodontitis	0.0097	0.011
UKB	Ulcers	0.082	0.0088
	Toothache	0.044	0.0072
	Bleeding gums	0.049	0.0033
	Painful gums	0.058	0.0098
	Dentures	0.094	0.0041
	Loose teeth	0.081	0.0091

Supplementary Table 18. Source data for Figure 1b (Estimated genetic correlation).

Trait 1	Trait 2	R_g	SE	p
DMFS	Ulcers	0.027	0.073	0.38
	Toothache	0.17	0.12	0.16
	Bleeding gums	-0.22	0.073	0.0029
	Painful gums	0.11	0.13	0.87
	Dentures	0.82	0.087	4.1x10-21
	Loose teeth	-0.008	0.090	0.93
DFSS	Ulcers	0.15	0.090	0.097
	Toothache	0.17	0.15	0.26
	Bleeding gums	-0.15	0.099	0.13
	Painful gums	-0.12	0.16	0.44
	Dentures	0.56	0.12	1.7x10-6
	Loose teeth	-0.37	0.13	0.0045
Nteeth	Ulcers	0.0057	0.060	0.92
	Toothache	-0.12	0.10	0.26
	Bleeding gums	0.036	0.071	0.51
	Painful gums	-0.11	0.11	0.34
	Dentures	-0.65	0.056	3.9x10-31
	Loose teeth	0.061	0.10	0.59
Periodontitis	Ulcers	0.011	0.19	0.95
	Toothache	0.44	0.48	0.93
	Bleeding gums	0.52	0.43	0.23
	Painful gums	0.24	0.39	0.54
	Dentures	0.51	0.42	0.23
	Loose teeth	1.07	0.78	0.17

Supplementary Note 1: Acknowledgements

ARIC

The Atherosclerosis Risk in Communities Study was carried out as a collaborative study supported by National Heart, Lung and Blood Institute contracts (HHSN268201100005C, HHSN268201100006C, HHSN268201100007C, HHSN268201100008C, HHSN268201100009C, HHSN268201100010C, HHSN268201100011C and HSN268201100012C), R01HL087641, R01HL59367 and R01HL086694; National Human Genome Research Institute contract U01HG004402; National Institutes of Health contract HHSN268200625226C; National Institute of Environmental Health Sciences grant P30ES010126; and National Institute of Dental and Craniofacial Research grants R01DE11551, R01DE021418 and R01DE023836. Infrastructure was partly supported by Grant Number UL1RR025005, a component of the National Institutes of Health and NIH Roadmap for Medical Research NCAT grant UL1-RR025747. KD and CA are partially supported by U01-DE025046.

COHRA1

All study protocols in COHRA were approved by the Institutional Review Board at the University of Pittsburgh. Analysis and genotyping in COHRA were funded through NIH grants U01-DE018903, R01-DE014899, R03-DE021425 and R03-DE024264, R56-DE027055. Genotyping was performed as part of the GENEVA consortium by the Center for Inherited Disease Research (www.cidr.jhmi.edu) through an NIH contract. Genome-wide summary statistics are available through the Human Genomics Analysis Interface of the FaceBase consortium (URL: http://FaceBase.sdmgenetics.pitt.edu/, NIH Grant # 5U01-DE024425).

DRDR

All study protocols in DRDR were approved by the Institutional Review Board at the University of Pittsburgh. The Dental Registry and DNA Repository is supported by the University of Pittsburgh. This work was funded through NIH grant U01-DE018903. Genotyping was performed as part of the GENEVA consortium by the Center for Inherited Disease Research (www.cidr.jhmi.edu) through an NIH contract.

HCHS/SOL

We thank the participants and staff of the Hispanic Community Health Study/Study of Latinos (HCHS/SOL) for their contributions to this study. The Hispanic Community Health Study/Study of Latinos was carried out as a collaborative study supported by contracts from the National Heart, Lung and Blood Institute (NHLBI) to the University of North Carolina (N01- HC65233), University of Miami (N01-HC65234), Albert Einstein College of Medicine (N01-HC65235), Northwestern University (N01-HC65236) and San Diego State University (N01-HC65237). The following Institutes/Centers/Offices contribute to the HCHS/SOL through a transfer of funds to the NHLBI: National Institute on Minority Health and Health Disparities, National Institute on Deafness and Other Communication Disorders, National Institute of Dental and Craniofacial Research, National Institute of Diabetes and Digestive and Kidney Diseases, National Institute of Neurological Disorders and Stroke, NIH Institution-Office of Dietary Supplements. The Genetic Analysis Center at the University of Washington was supported by NHLBI and NIDCR contracts (HHSN268201300005C AM03 and MOD03). KG was supported by the National Science Foundation Graduate Research Fellowship Program under Grant No. DGE-1256082. Any opinions, findings and conclusions or recommendations expressed in this material are those of the author(s) and do not necessarily reflect the views of the National Science Foundation.

MDC

The Malmö Diet Cancer study has received support from a number of sources including the Swedish Research Council, Swedish Heart-Lung Foundation, Swedish Cancer Foundation Albert Pahlsson Foundation, Lundströms Foundation and the city of Malmö.

NFBC1966

We thank the late Professor Paula Rantakallio (launch of NFBC1966), the participants in the 46y study and the NFBC project center. NFBC1966 received financial support from University of Oulu Grant no. 24000692, Oulu University Hospital Grant no. 24301140 and ERDF European Regional Development Fund Grant no. 539/2010 A31592. The authors would also like to thank Mr. Jari Päkkilä for designing the software (electronic patient file) used in NFBC1966.

SHIP and *SHIP-TREND*

SHIP is part of the Community Medicine Research net (CMR http://www.community-medicine.de) of the University of Greifswald, Germany, which is funded by the Federal Ministry of Education and Research (grants no. 01ZZ9603, 01ZZ0103 and 01ZZ0403), the Ministry of Cultural

Affairs as well as the Social Ministry of the Federal State of Mecklenburg-West Pomerania. Generation of genome-wide SNP data has been supported by the Federal Ministry of Education and Research (grant no. 03ZIK012) and a joint grant from Siemens Healthcare, Erlangen, Germany and the Federal State of Mecklenburg, West Pomerania. The University of Greifswald is a member of the Caché Campus programme of the InterSystems GmbH.

TWINGENE

TwinGene is a substudy of the Swedish Twin Registry which is managed by Karolinska Institutet and receives funding through the Swedish Research Council under the grant no 2017-00641.

WGHS

The WGHS is supported by the National Heart, Lung, and Blood Institute (HL043851 and HL080467) and the National Cancer Institute (CA047988 and UM1CA182913) with funding for genotyping from Amgen. Yu YH is supported by the National Institute of Dental and Craniofacial Research (1K23DE026804-01A1).

BBJ

The Biobank Japan project was supported by the Ministry of Education, Culture, Sports, Science, and Technology, Japanese government and the Japan Agency for Medical Research and Development.

TMDUAGP

The Tokyo Medical and Dental University Aggressive Periodontitis Study received support from the Japan Society for the Promotion of Science.

Swedish GLIDE

The Swedish GLIDE project is funded by the Swedish Research Council (Dnr 2011-3372 and 2015-02597), the Västerbotten County Council and Umeå University, Sweden. NJT is a Wellcome investigator (202802/Z/16/Z), a work-package lead in the Integrative Cancer Epidemiology Programme (ICEP) that is supported by a Cancer Research UK programme grant (C18281/A19169) and works within the University of Bristol NIHR Biomedical Research Centre (BRC). DS is supported by the Swedish Research Council (4.1-2016-00416). SH receives support from Wellcome (Grant ref: 201237/Z/16/Z). This work was carried out using the computation facilities of the Advanced Computing Research Centre - http://www.bris.ac.uk/acrc/, the Research Data Storage Facility of the University of Bristol - http://www.bris.ac.uk/acrc/storage/ and the High Performance Computing Center North (HPC2N) at Umeå University – http://www.hpc2n.umu.se.

External resources

The LDHub and PhenoScanner resources are made possible by studies and databases which made GWAS summary data available. These are listed in full online at (http://ldsc.broadinstitute.org/about/) and (http://www.phenoscanner.medschl.cam.ac.uk/information.html).

LDHub gratefully acknoleges the contributions of ADIPOGen (Adiponectin genetics consortium), C4D (Coronary Artery Disease Genetics Consortium), CARDIoGRAM (Coronary ARtery DIsease Genome wide Replication and Meta-analysis), CKDGen (Chronic Kidney Disease Genetics consortium), dbGAP (database of Genotypes and Phenotypes), DIAGRAM (DIAbetes Genetics Replication And Meta-analysis), ENIGMA (Enhancing Neuro Imaging Genetics through Meta Analysis), EAGLE (EArly Genetics & Lifecourse Epidemiology Eczema Consortium, excluding 23andMe), EGG (Early Growth Genetics Consortium), GABRIEL (A Multidisciplinary Study to Identify the Genetic and Environmental Causes of Asthma in the European Community), GCAN (Genetic Consortium for Anorexia Nervosa), GEFOS (GEnetic Factors for OSteoporosis Consortium), GIANT (Genetic Investigation of ANthropometric Traits), GIS (Genetics of Iron Status consortium), GLGC (Global Lipids Genetics Consortium), GPC (Genetics of Personality Consortium), GUGC (Global Urate and Gout consortium), HaemGen (haemotological and platelet traits genetics consortium), HRgene (Heart Rate consortium), IIBDGC (International Inflammatory Bowel Disease Genetics Consortium), ILCCO (International Lung Cancer Consortium), IMSGC (International Multiple Sclerosis Genetic Consortium), MAGIC (Meta-Analyses of Glucose and Insulin-related traits Consortium), MESA (Multi-Ethnic Study of Atherosclerosis), PGC (Psychiatric Genomics Consortium), Project MinE consortium, ReproGen (Reproductive Genetics Consortium), SSGAC (Social Science Genetics Association Consortium) and TAG (Tobacco and Genetics Consortium), TRICL (Transdisciplinary Research in Cancer of the Lung consortium), UK Biobank., Alkes Price (the systemic lupus erythematosus GWAS and primary biliary cirrhosis GWAS) and Johannes Kettunen (lipids metabolites GWAS).

We are grateful to GIANT, ENGAGE, DIAGRAM, GLGC, MEGASTROKE and CARDIoGRAMplusC4D consortia for making data available on metabolic traits and cardiovascular outcomes used in the MR analysis included in this manuscript.

Specifically, data were downloaded from the following sources;
GIANT consortium (adiposity traits);
https://portals.broadinstitute.org/collaboration/giant/index.php/GIANT_consortium_data_files
The ENGAGE consortium (fasting glucose); http://mccarthy.well.ox.ac.uk/publications/2015/ENGAGE_1KG/.
The DIAGRAM consortium (type 2 diabetes); http://www.diagram-consortium.org/downloads.html.
GLGC (lipid traits); http://csg.sph.umich.edu/willer/public/lipids2013/.
MEGASTROKE (all stroke); http://megastroke.org/download.html.
CARDIoGRAMplusC4D (coronary artery disease/myocardial infarction); www.CARDIOGRAMPLUSC4D.ORG..
The MEGASTROKE project received funding from sources specified at http://megastroke.org/acknowlegements.html

Supplementary Note 2: Standardized regression coefficients.

Standardized regression coefficients were derived from Z-scores and effect allele frequencies using the following transformation;

$$\widehat{\beta_J} \approx Z_j \frac{\widehat{\sigma_y}}{\sqrt{N_j \times 2(1 - EAF_j)EAF_j}}$$

where SNP j has an effect allele frequency (EAF_j) and $\widehat{\sigma_y}$ is standard deviation of the phenotype, which was arbitrarily set at 1. The standard error (SE) was calculated as

$$Z_j = \frac{\widehat{\beta_J}}{SE(\widehat{\beta_J})}$$

Supplementary Note 3: Indicative effect sizes

First, beta coefficients in the clinical analysis (β_{j_GLIDE}) were regressed on the standardized (β_j) in a regression including all conditionally-independent markers passing genome-wide significance (for DMFS/dentures) and all conditionally-independent markers with $p<1x10^{-5}$ for periodontitis/loose teeth. The gradient of these regression models was used to rescale standardized regression coefficients to the same scale used in GLIDE, now interpreted as an approximation of standard deviations of inverse normal transformed DMFS residuals or log odds ratios (for periodontitis/loose teeth).

Finally, to relate effect sizes in the transformed DMFS variable to absolute effect sizes (tooth surfaces), phenotype preparation was repeated in an independent population-representative collection of clinically obtained dental data. Transformed z scores were created for 28,691 adults (aged 30-75 years) with clinical dental examination data in the Swedish GLIDE database, who were originally recruited from the population-based Northern Sweden Health and Disease study[1]. Raw DMFS counts were regressed on the transformed z score, estimating the number of tooth surfaces corresponding to a 1-unit change at the population-level mean level of dental caries experience and covariates.

Supplementary Note 4: Mendelian randomization sensitivity analyses.

For sensitivity analysis, GSMR results were compared to results from standard SNP-based two-sample estimation approaches using GSMR to select instruments and harmonize summary statistics but omitting the HEIDI test. After harmonization, summary statistics were imported into R for sensitivity analysis. Inverse Variance Weighted Meta-analysis and MR-Egger[2] tests were performed using the 'MendelianRandomization' R package (v0.3), and a model averaging procedure using a heterogeneity-penalized modal estimator was performed using R code supplied in manuscript which introduces the method[3]. Where more than one exposure was nominally associated with the same outcome, multivariable MR was performed using the MV IVW estimator[4], also implemented in the 'MendelianRandomization' R package.

Supplementary references

1. Hallmans, G. *et al.* Cardiovascular disease and diabetes in the Northern Sweden Health and Disease Study Cohort - evaluation of risk factors and their interactions. *Scan. J. Pub. Health. Supp.* **61**, 18-24 (2003).
2. Bowden, J., Davey Smith, G. & Burgess, S. Mendelian randomization with invalid instruments: effect estimation and bias detection through Egger regression. *Int. J. Epidemiol* **44**, 512-525 (2015).
3. Burgess, S., Zuber, V., Gkatzionis, A. & Foley, C.N. Modal-based estimation via heterogeneity-penalized weighting: model averaging for consistent and efficient estimation in Mendelian randomization when a plurality of candidate instruments are valid. *Int. J. Epidemiol.*, dyy080-dyy080 (2018).
4. Burgess, S. & Thompson, S.G. Multivariable Mendelian randomization: the use of pleiotropic genetic variants to estimate causal effects. *Am. J. Epidemiol.* **181**, 251-260 (2015).
