# Supplementary material for: Genome-wide analysis of dental caries and periodontitis combining clinical and self-reported data
Source: Nat Commun. 2019 Jun 24;10:2773. doi: 10.1038/s41467-019-10630-1 (PMC6591304; doi:10.1038/s41467-019-10630-1)
Supplement: Supplementary file 3 — Description of Additional Supplementary Files [file 41467_2019_10630_MOESM3_ESM.docx]

**Title:** Supplementary Data 1
**Description:** Description of cohorts participating in analysis

**Title:** Supplementary Data 2
**Description:** Description of genotyping, quality control and statistical analysis

**Title:** Supplementary Data 3
**Description:** Lead variants for novel risk loci identified in DMFS/dentures combined metaanalysis

**Title:** Supplementary Data 4
**Description:** Results of PhenoScanner cross-trait single variant lookup of DMFS/dentures loci

**Title:** Supplementary Data 5
**Description:** Regional association plots for novel risk loci identified in DMFS/dentures combined meta-analysis

**Title:** Supplementary Data 6
**Description:** TissueXcan results for combined analysis of DMFS/dentures passing a Bonferroni correction

**Title:** Supplementary Data 7
**Description:** Full TissueXcan results for combined analysis of DMFS/dentures

**Title:** Supplementary Data 8
**Description:** Full TissueXcan results for combined analysis of periodontitis/loose teeth

**Title:** Supplementary data 9
**Description:** DEPICT gene set enrichment analysis for combined analysis of DMFS/dentures

**Title:** Supplementary Data 10
**Description:** Heritability of DMFS/dentures partitioned by functional annotations

**Title:** Supplementary Data 11
**Description:** Heritabiltiy of DMFS/dentures partitioned by tissue-specific annotations

**Title:** Supplementary Data 12
**Description:** Heritabiltiy of periodontitis/ loose teeth partitioned by functional annotations

**Title:** Supplementary Data 13
**Description:** Heritability of periodontitis/loose teeth stratified by tissue-specific annotation

**Title:** Supplementary Data 14
**Description:** Results of hypothesis-free cross-trait genetic correlation analysis for DMFS/Dentures combined analysis

**Title:** Supplementary Data 15
**Description:** Results of cross-trait hypothesis free genetic correlation analysis for periodontitis / loose teeth combined analysis
