# Supplementary figures and images for: Genome-wide analysis of dental caries and periodontitis combining clinical and self-reported data
Source: Nat Commun. 2019 Jun 24;10:2773. doi: 10.1038/s41467-019-10630-1 (PMC6591304; doi:10.1038/s41467-019-10630-1)

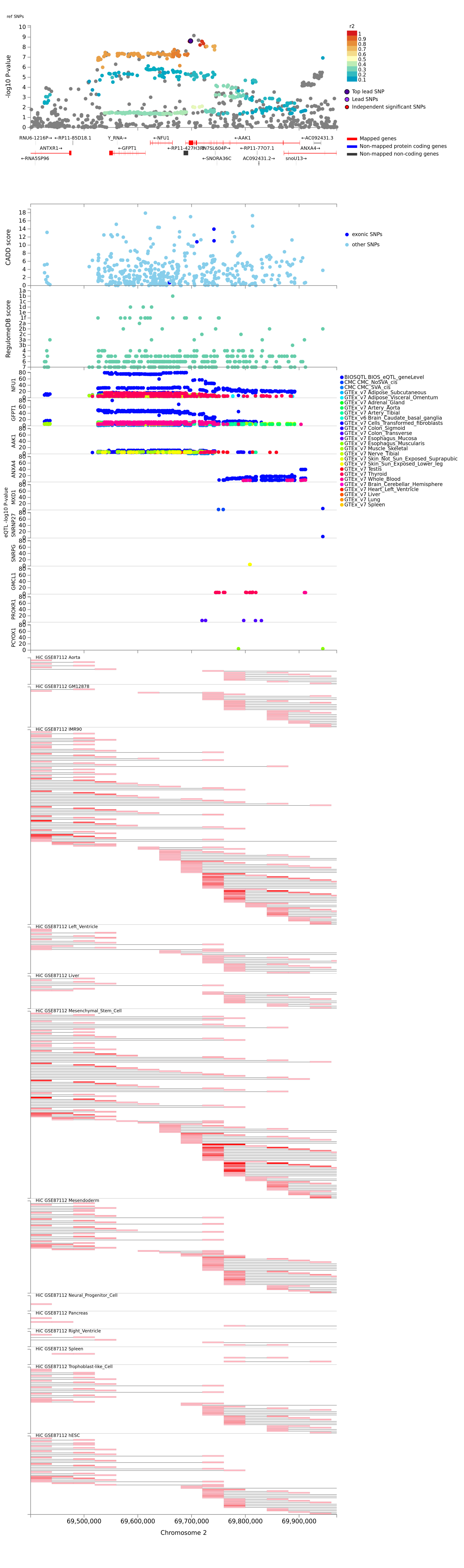

Supplement: Supplementary file 8 — Supplementary Dataset 5 [file 41467_2019_10630_MOESM8_ESM.zip › regional_association/AAK1.pdf]

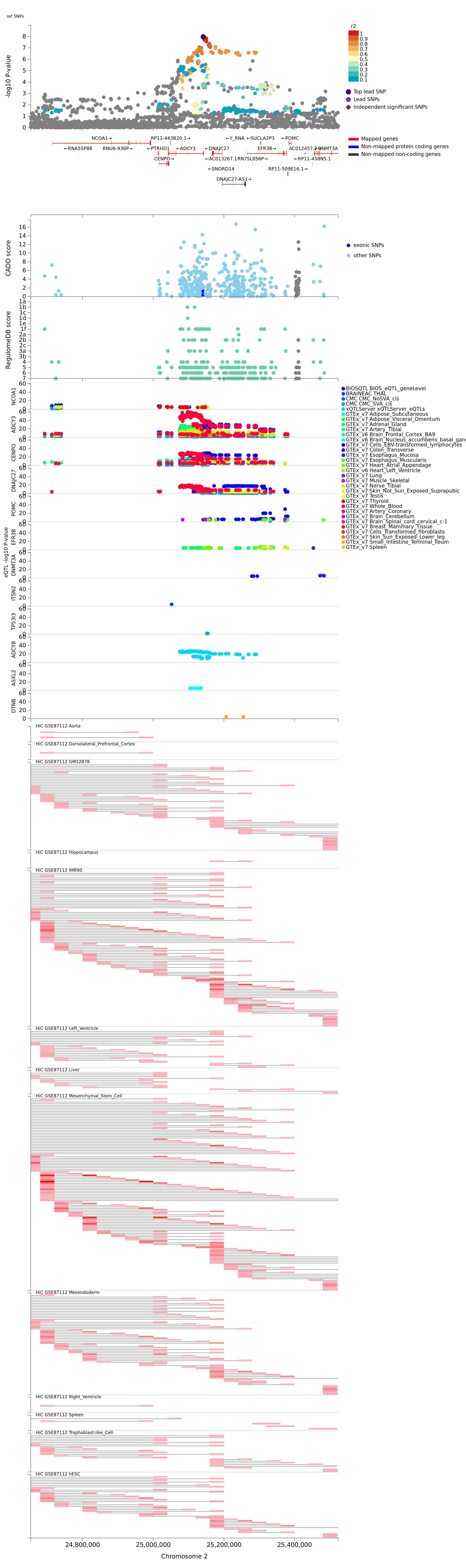

Supplement: Supplementary file 8 — Supplementary Dataset 5 [file 41467_2019_10630_MOESM8_ESM.zip › regional_association/ADCY3.pdf]

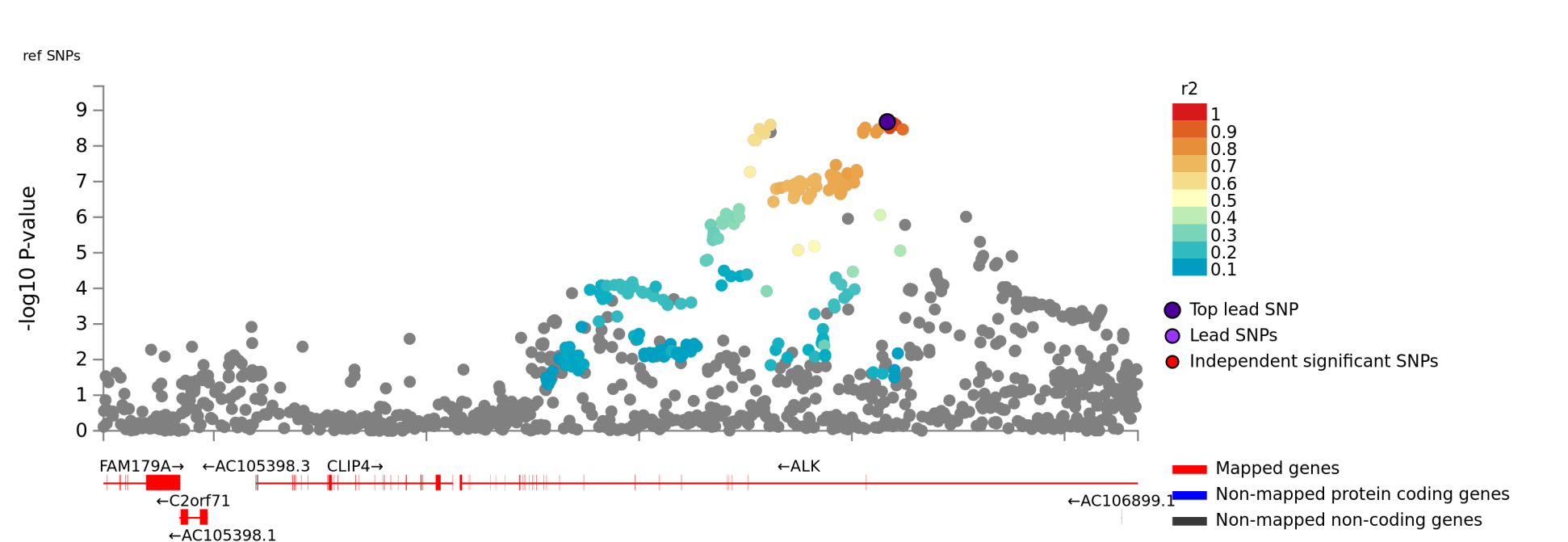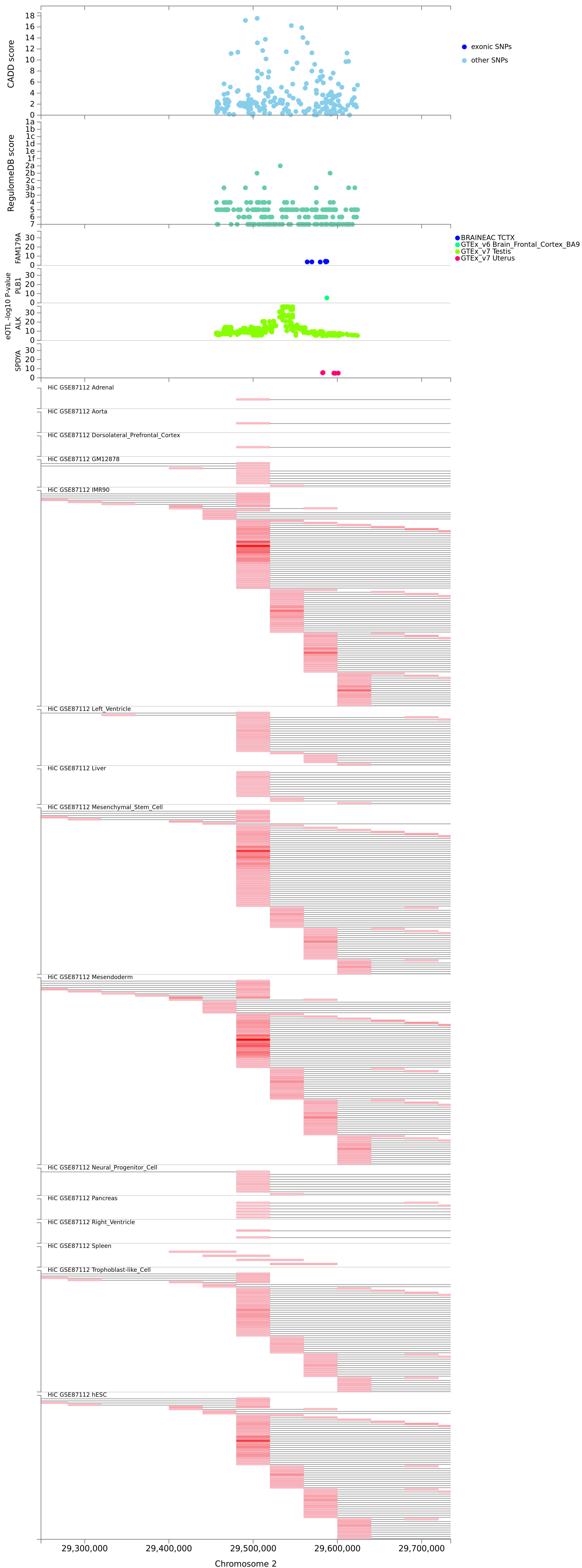

Supplement: Supplementary file 8 — Supplementary Dataset 5 [file 41467_2019_10630_MOESM8_ESM.zip › regional_association/ALK.pdf]

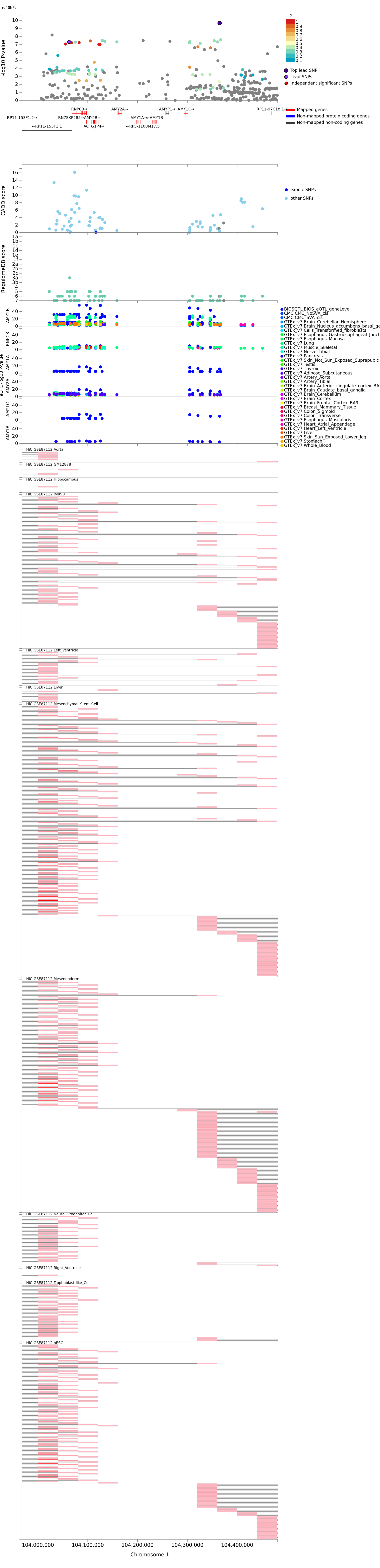

Supplement: Supplementary file 8 — Supplementary Dataset 5 [file 41467_2019_10630_MOESM8_ESM.zip › regional_association/AMY1C.pdf]

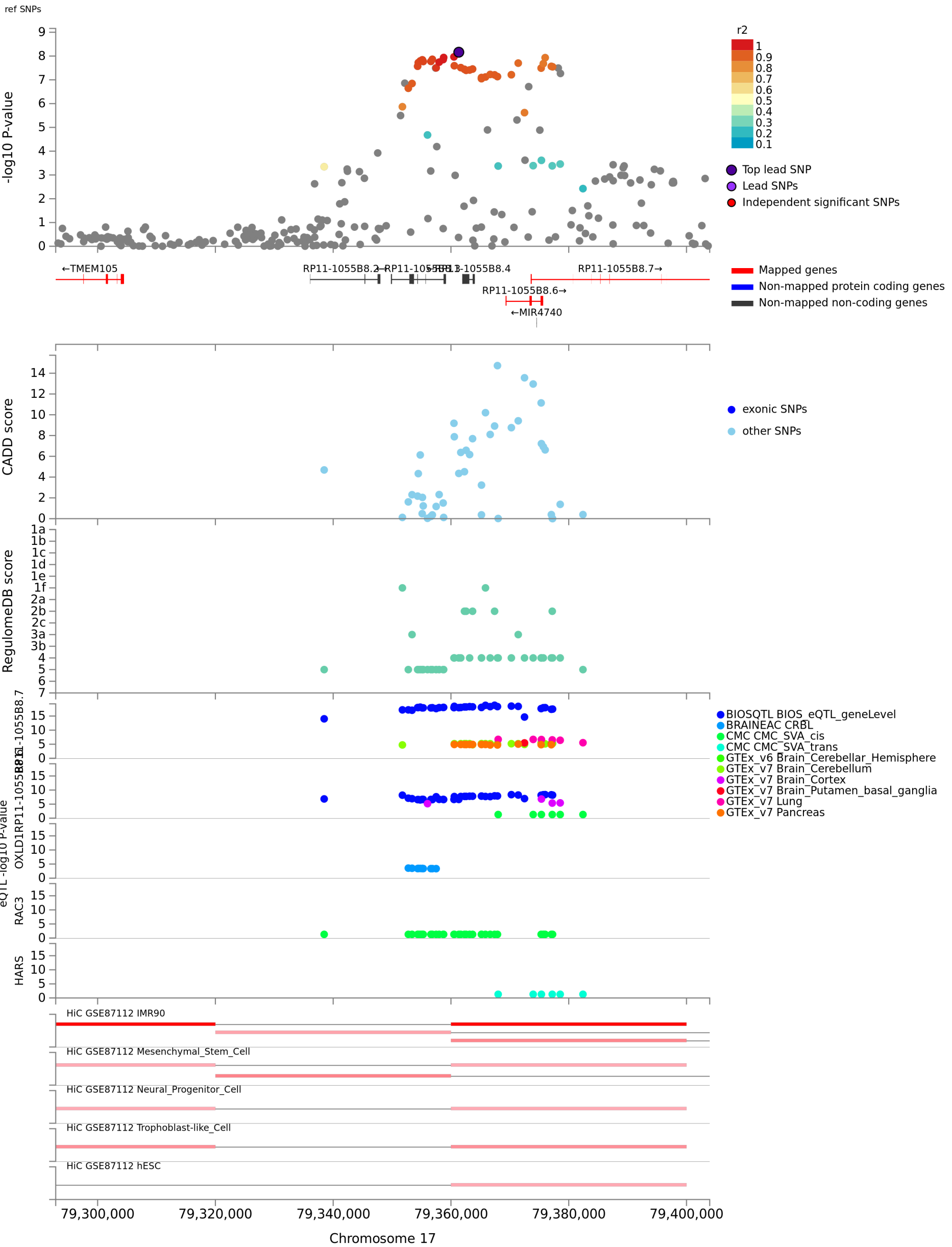

Supplement: Supplementary file 8 — Supplementary Dataset 5 [file 41467_2019_10630_MOESM8_ESM.zip › regional_association/BAHCC1.pdf]

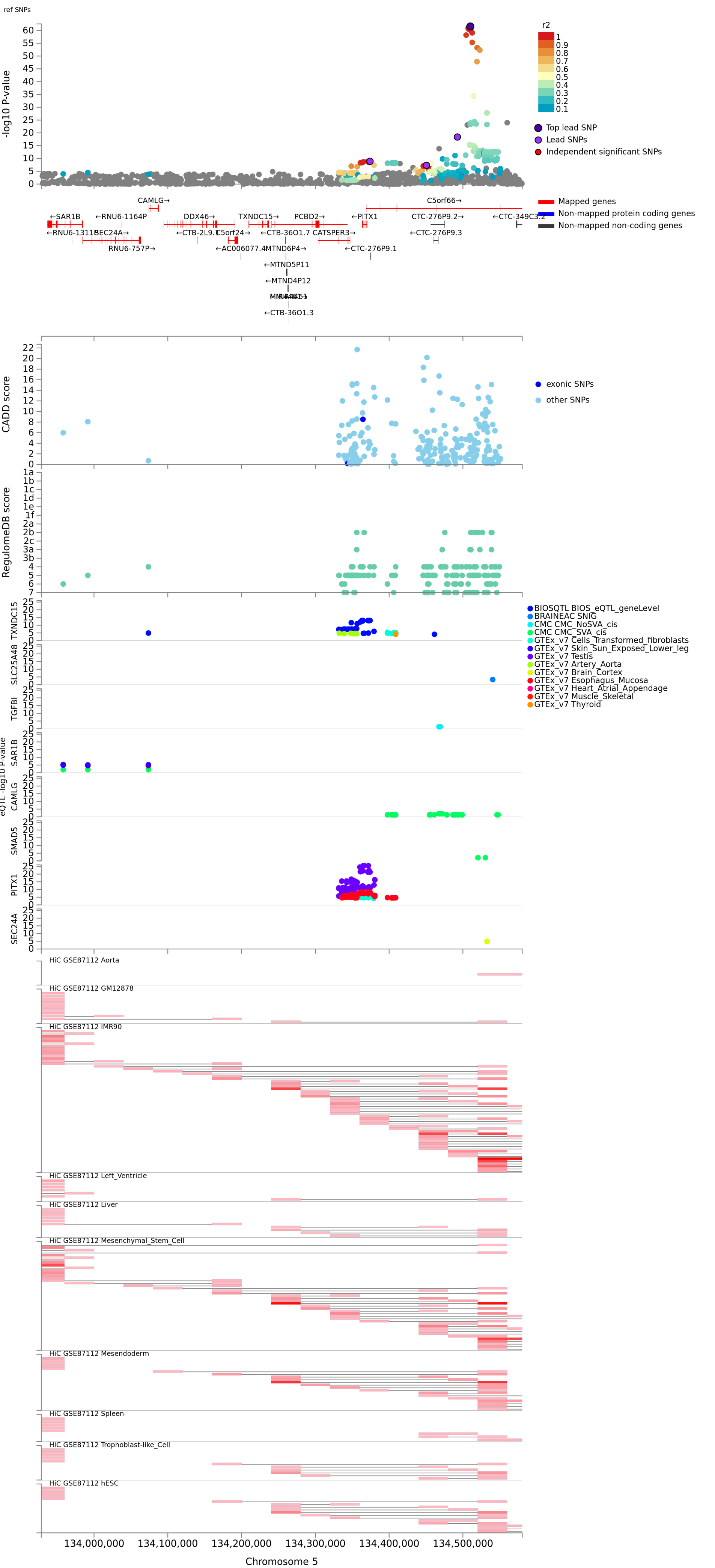

Supplement: Supplementary file 8 — Supplementary Dataset 5 [file 41467_2019_10630_MOESM8_ESM.zip › regional_association/C5orf66.pdf]

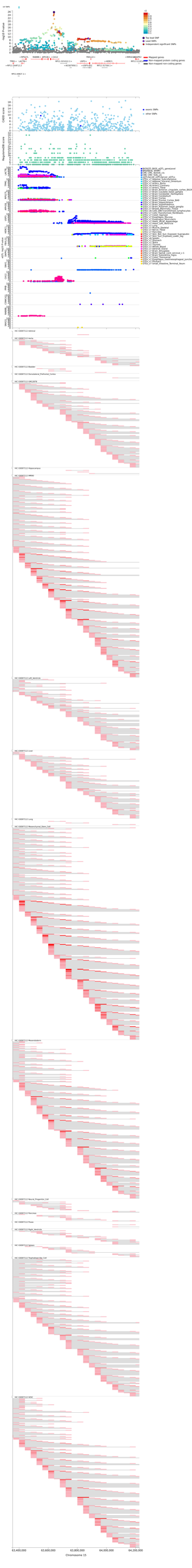

Supplement: Supplementary file 8 — Supplementary Dataset 5 [file 41467_2019_10630_MOESM8_ESM.zip › regional_association/CA12.pdf]

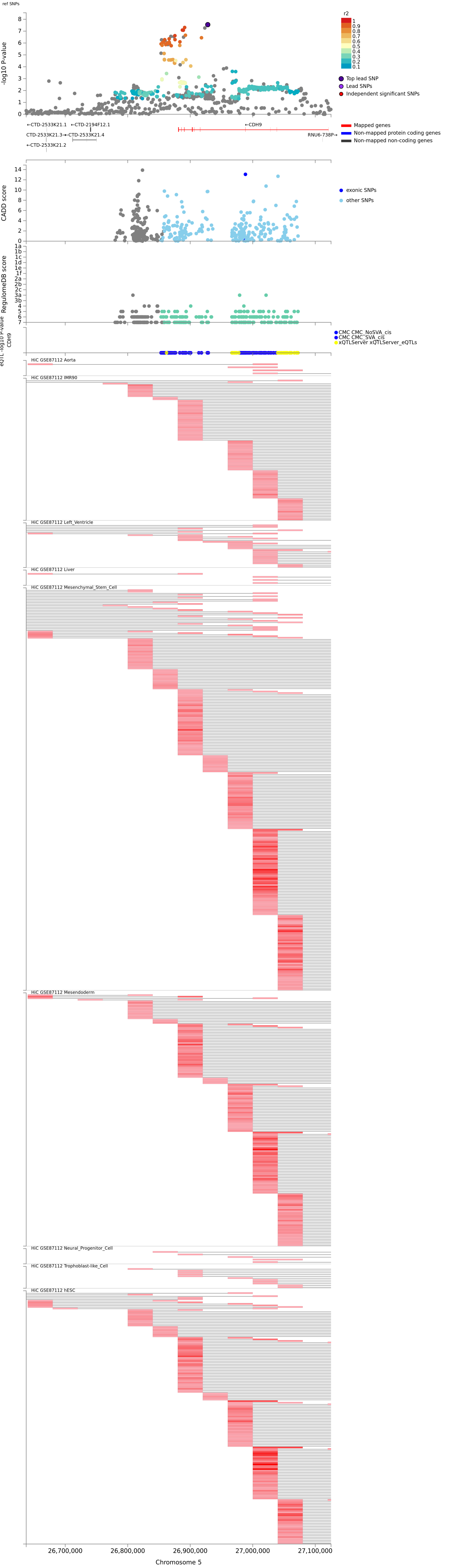

Supplement: Supplementary file 8 — Supplementary Dataset 5 [file 41467_2019_10630_MOESM8_ESM.zip › regional_association/CDH9.pdf]

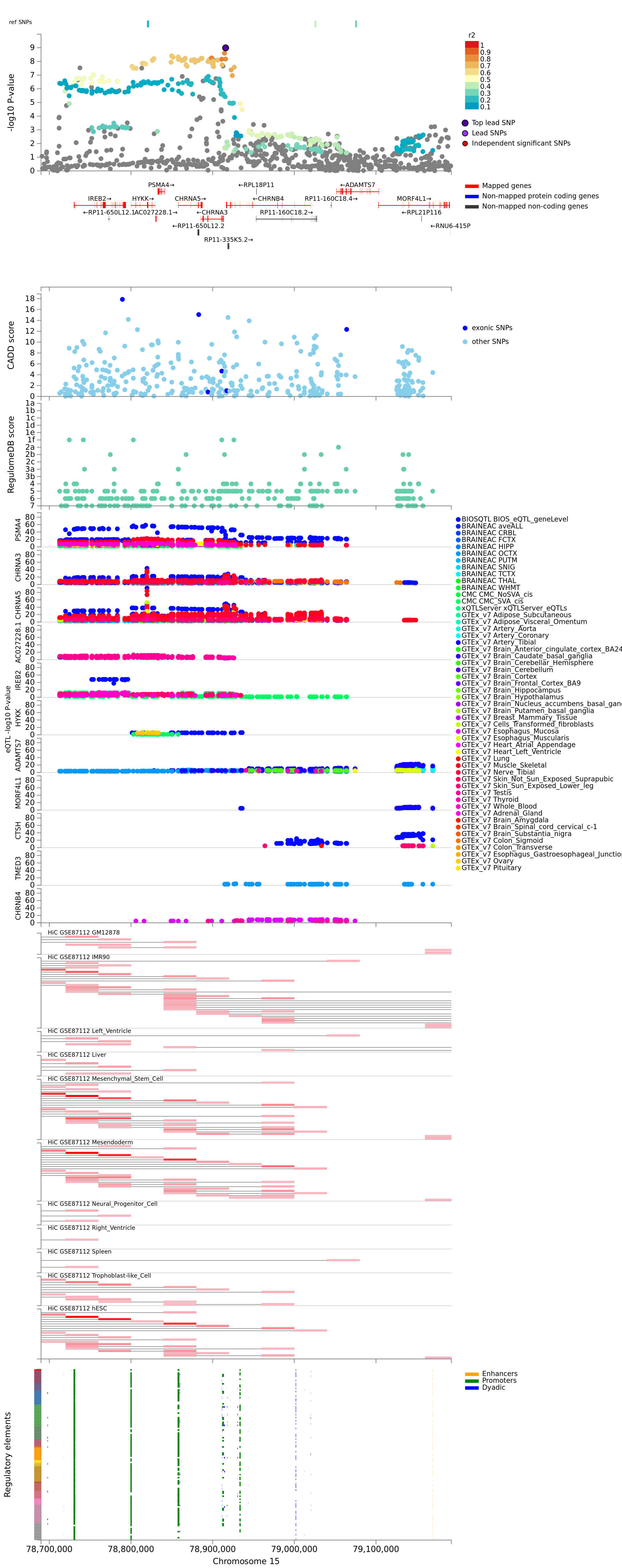

Supplement: Supplementary file 8 — Supplementary Dataset 5 [file 41467_2019_10630_MOESM8_ESM.zip › regional_association/CHRNA3.pdf]

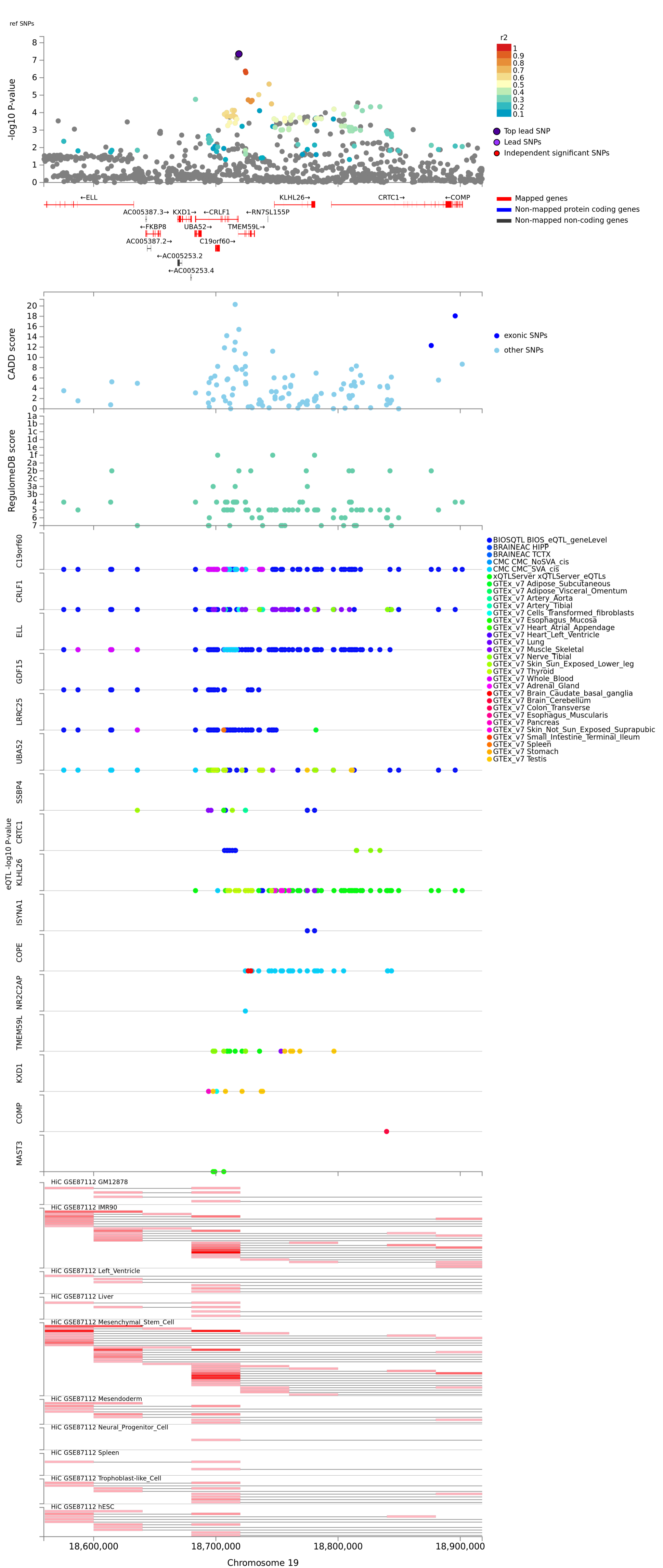

Supplement: Supplementary file 8 — Supplementary Dataset 5 [file 41467_2019_10630_MOESM8_ESM.zip › regional_association/CRLF1.pdf]

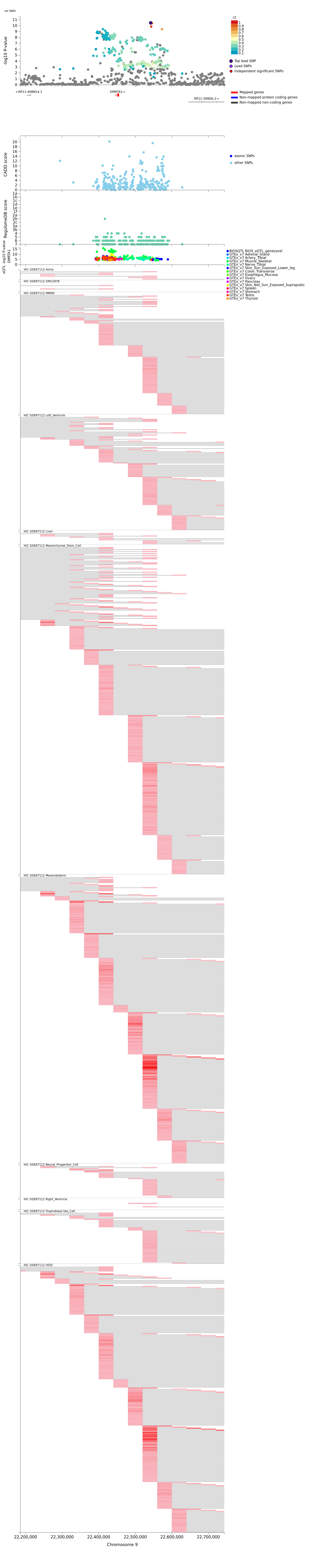

Supplement: Supplementary file 8 — Supplementary Dataset 5 [file 41467_2019_10630_MOESM8_ESM.zip › regional_association/DMRTA1.pdf]

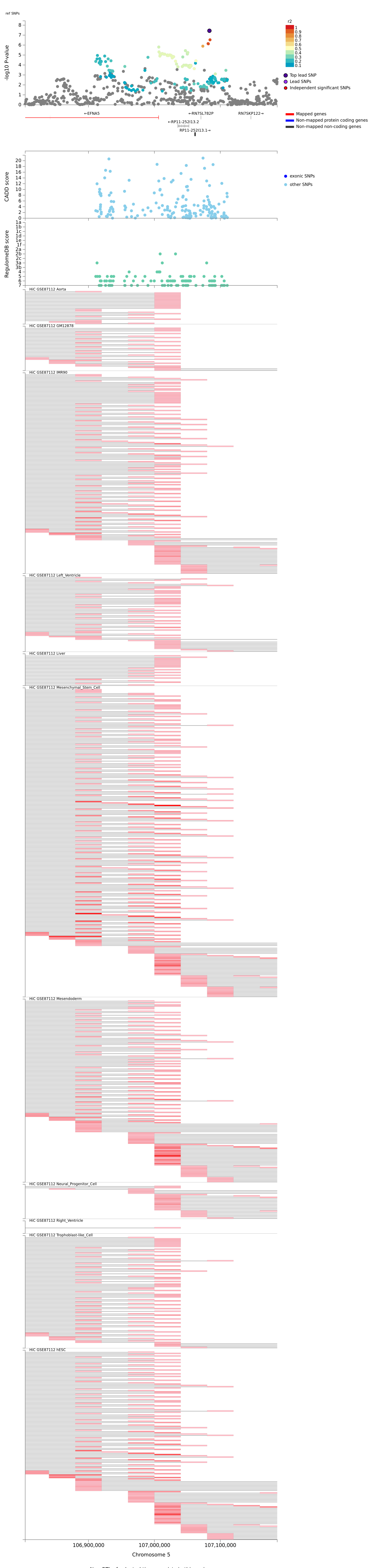

Supplement: Supplementary file 8 — Supplementary Dataset 5 [file 41467_2019_10630_MOESM8_ESM.zip › regional_association/EFNA5.pdf]

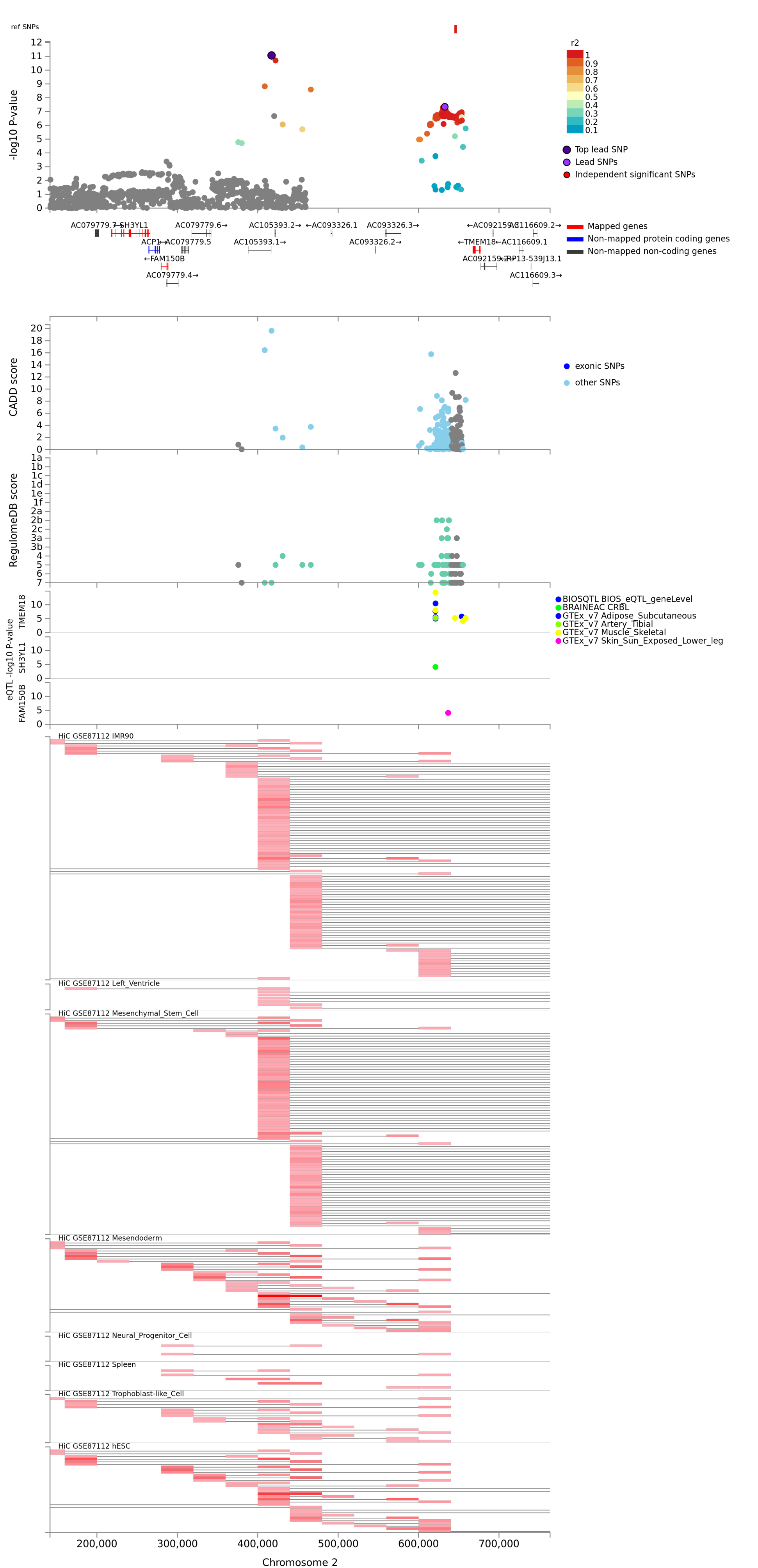

Supplement: Supplementary file 8 — Supplementary Dataset 5 [file 41467_2019_10630_MOESM8_ESM.zip › regional_association/FAM150B.pdf]

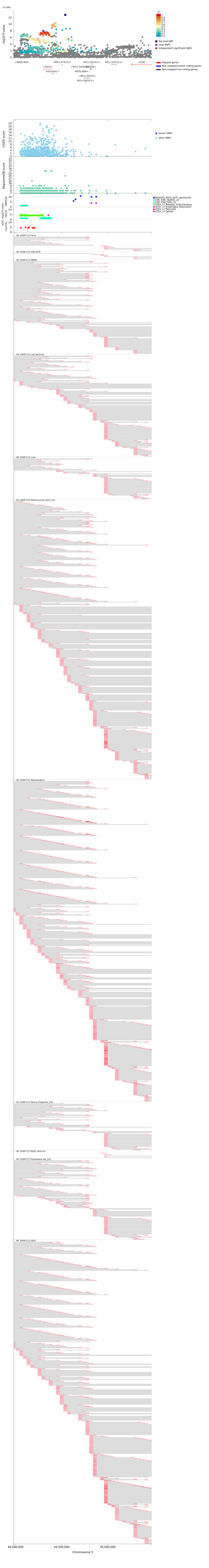

Supplement: Supplementary file 8 — Supplementary Dataset 5 [file 41467_2019_10630_MOESM8_ESM.zip › regional_association/FGF10.pdf]

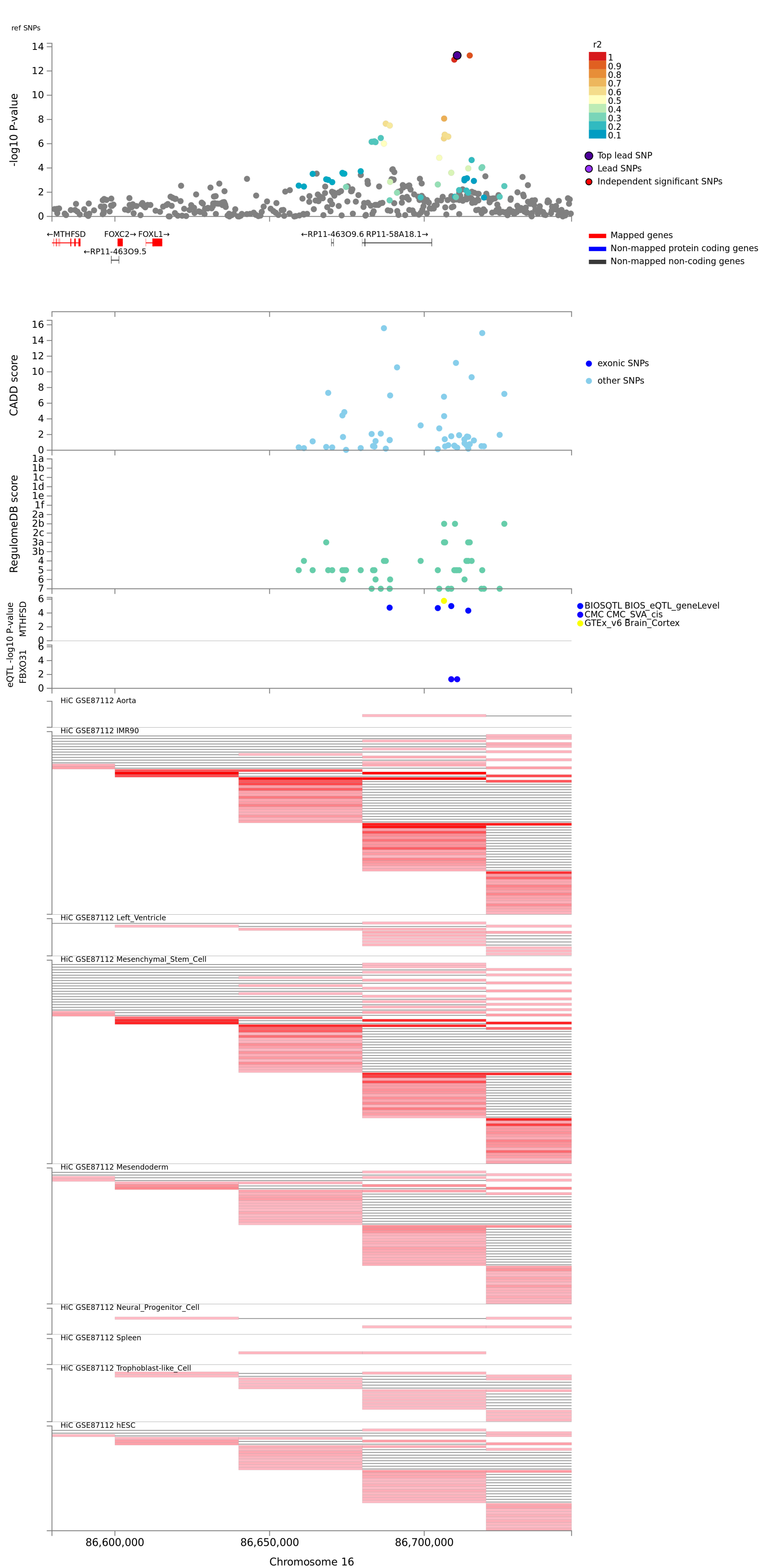

Supplement: Supplementary file 8 — Supplementary Dataset 5 [file 41467_2019_10630_MOESM8_ESM.zip › regional_association/FOXL1.pdf]

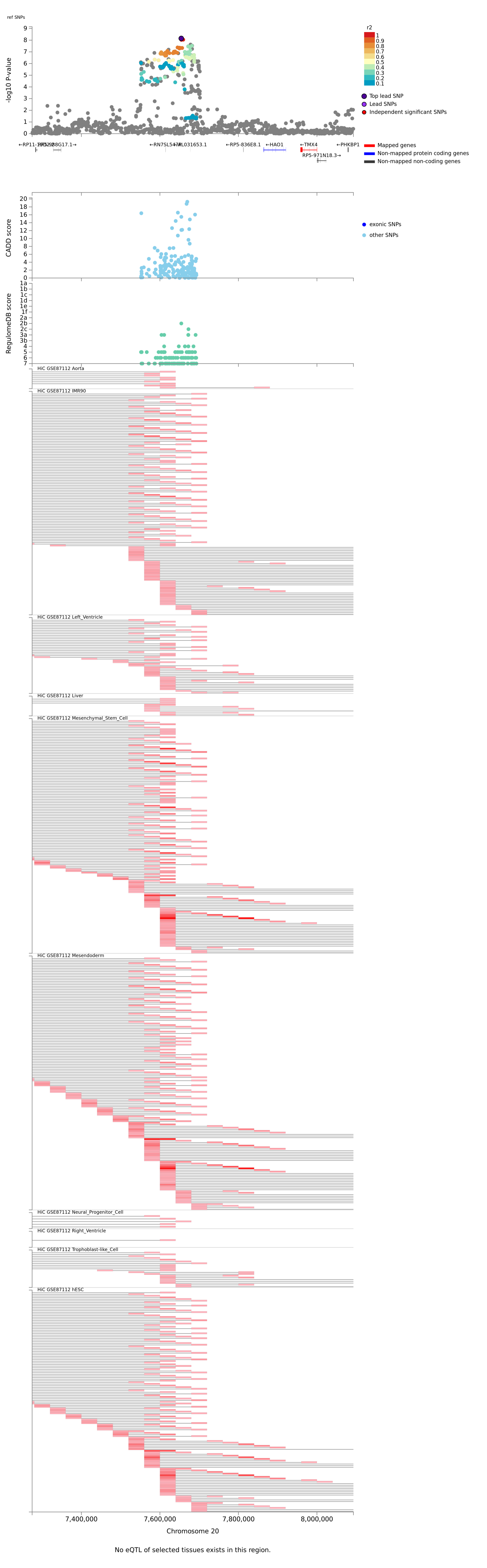

Supplement: Supplementary file 8 — Supplementary Dataset 5 [file 41467_2019_10630_MOESM8_ESM.zip › regional_association/HAO1.pdf]

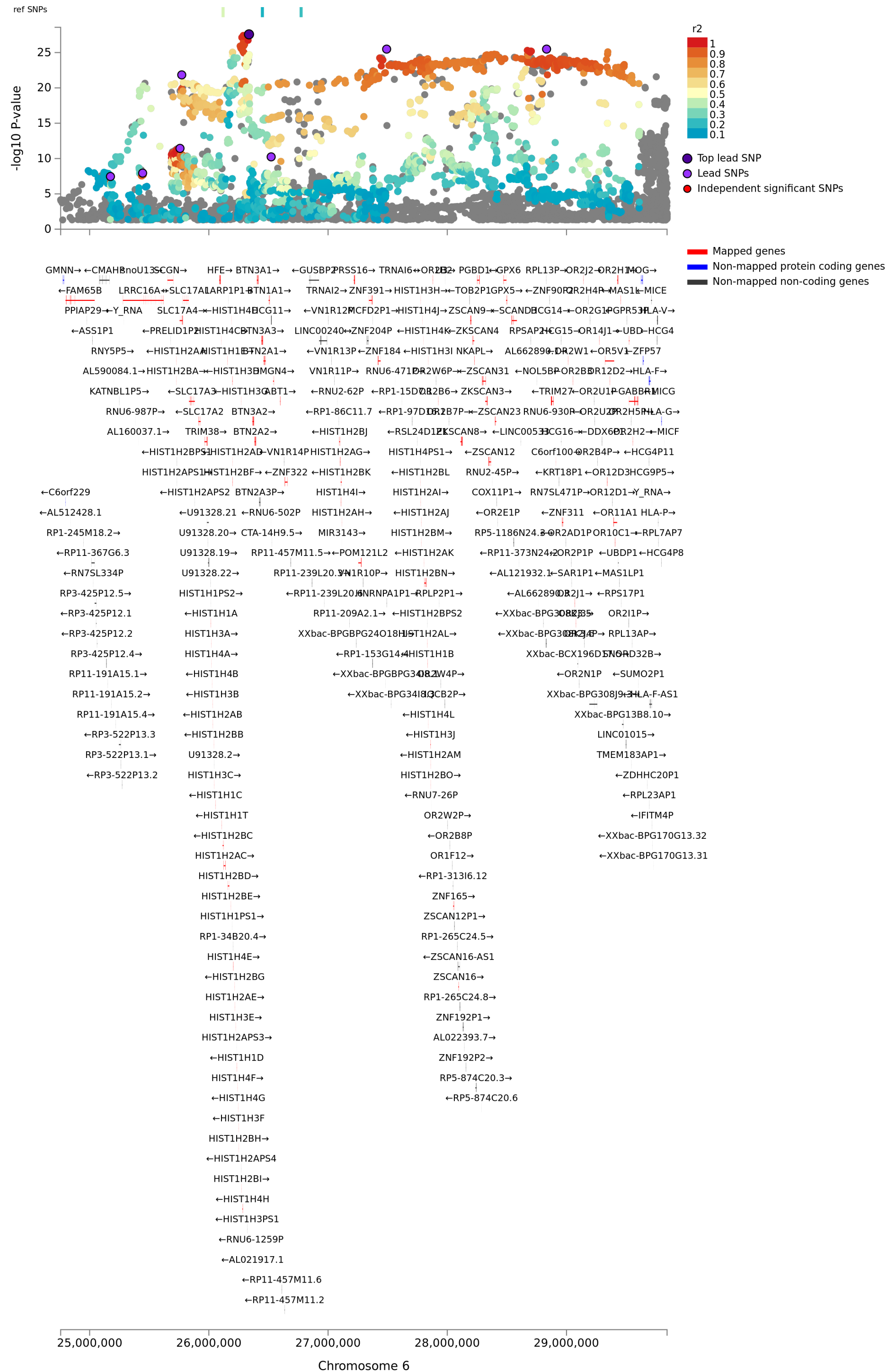

Supplement: Supplementary file 8 — Supplementary Dataset 5 [file 41467_2019_10630_MOESM8_ESM.zip › regional_association/HLA.pdf]

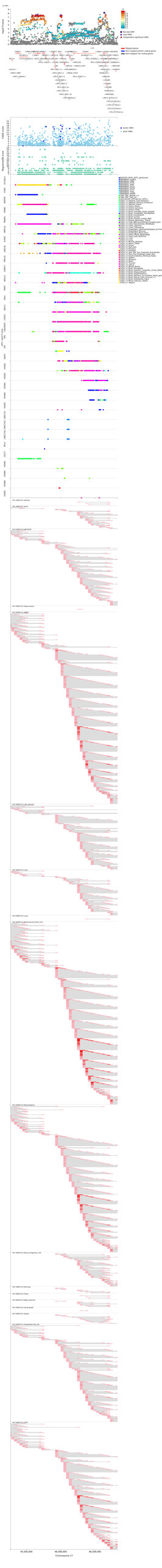

Supplement: Supplementary file 8 — Supplementary Dataset 5 [file 41467_2019_10630_MOESM8_ESM.zip › regional_association/HOSB-AS2_NPEPPS.pdf]

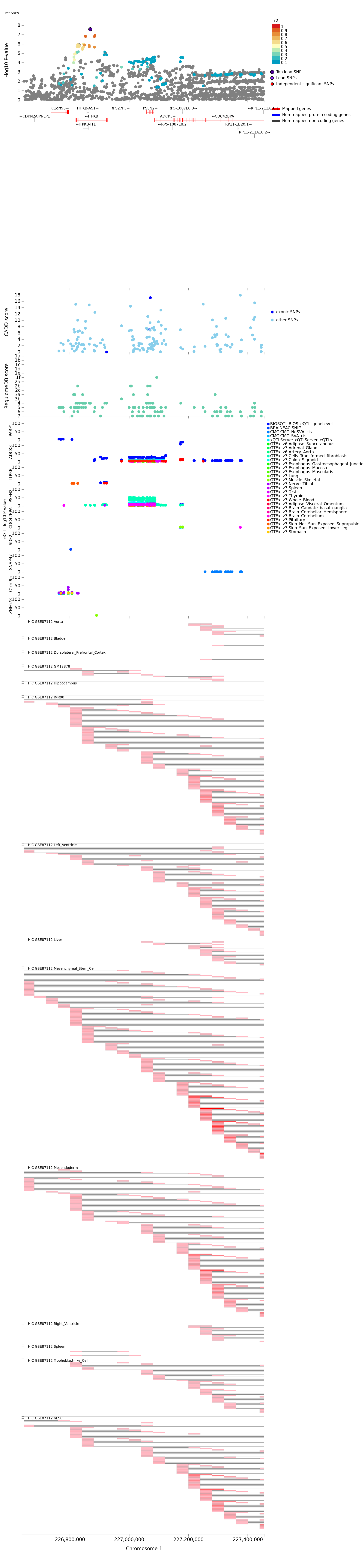

Supplement: Supplementary file 8 — Supplementary Dataset 5 [file 41467_2019_10630_MOESM8_ESM.zip › regional_association/ITPKB.pdf]

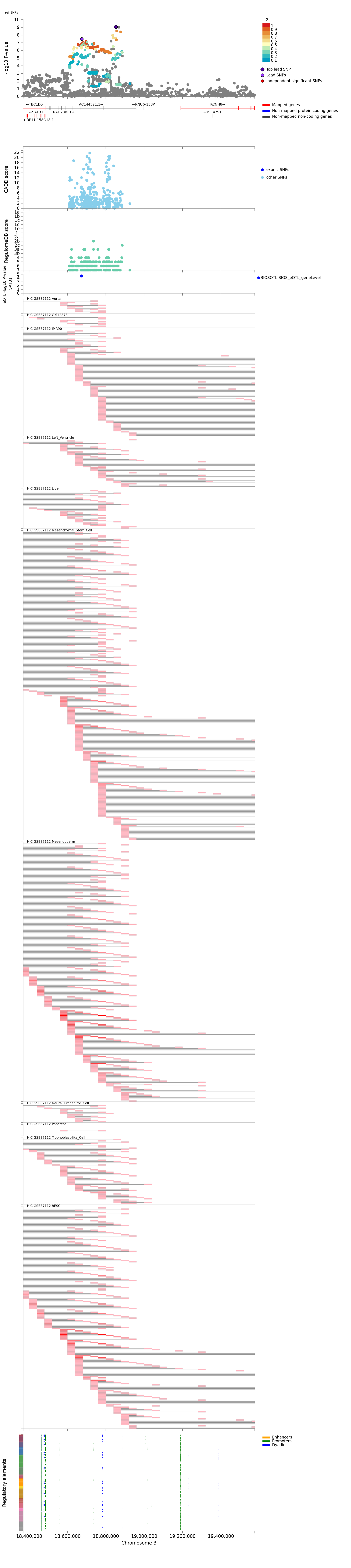

Supplement: Supplementary file 8 — Supplementary Dataset 5 [file 41467_2019_10630_MOESM8_ESM.zip › regional_association/KCNH8.pdf]

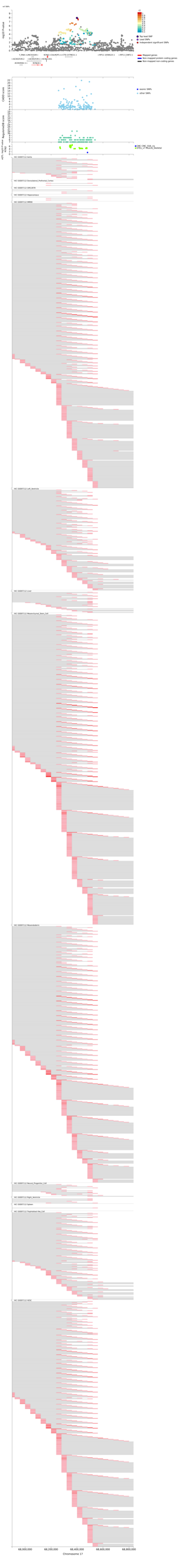

Supplement: Supplementary file 8 — Supplementary Dataset 5 [file 41467_2019_10630_MOESM8_ESM.zip › regional_association/KCNJ2.pdf]

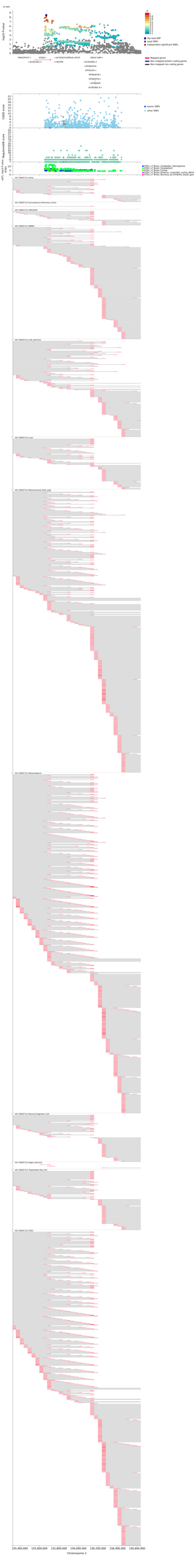

Supplement: Supplementary file 8 — Supplementary Dataset 5 [file 41467_2019_10630_MOESM8_ESM.zip › regional_association/KCNJ3.pdf]

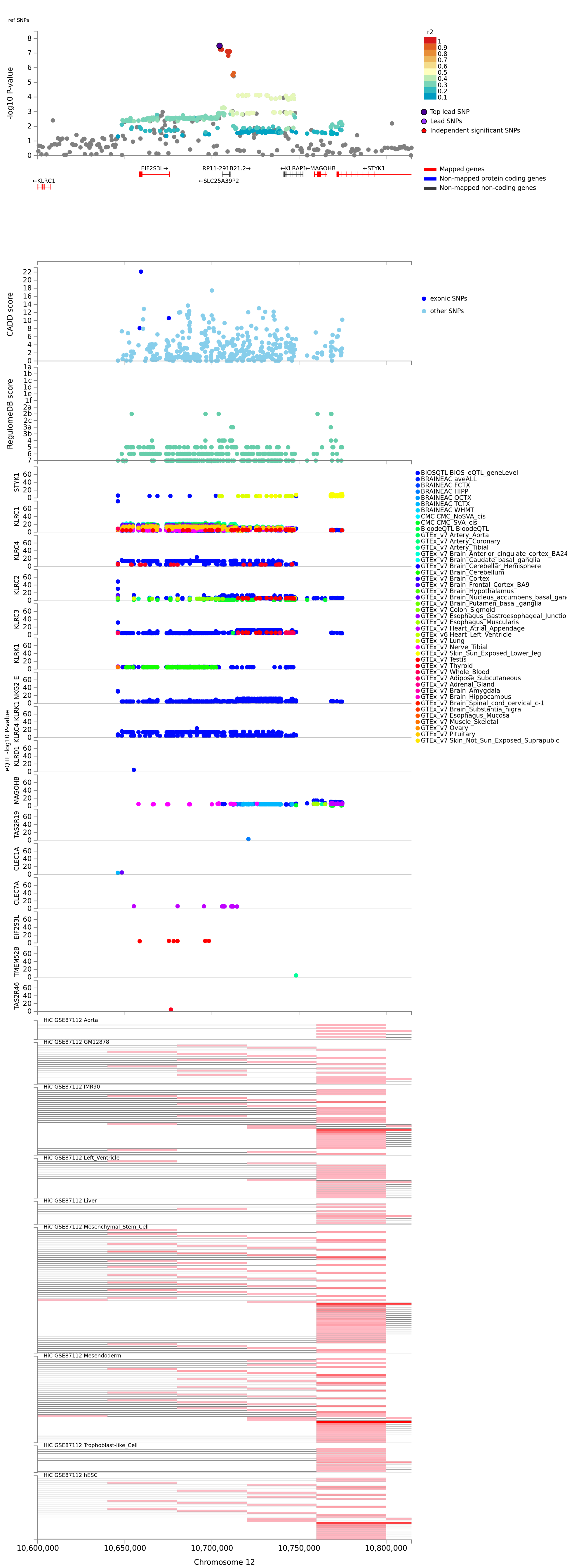

Supplement: Supplementary file 8 — Supplementary Dataset 5 [file 41467_2019_10630_MOESM8_ESM.zip › regional_association/KLRAP1.pdf]

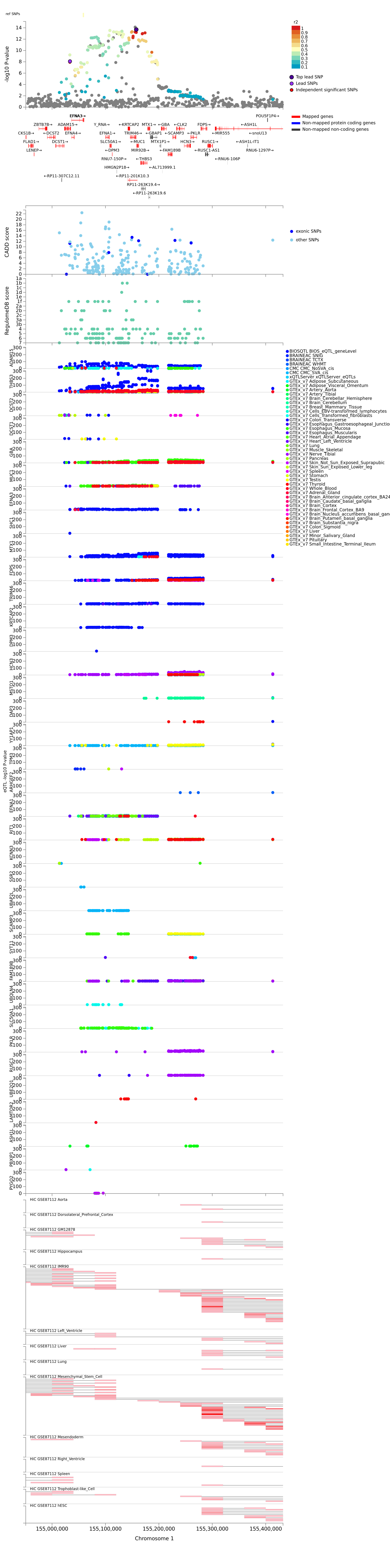

Supplement: Supplementary file 8 — Supplementary Dataset 5 [file 41467_2019_10630_MOESM8_ESM.zip › regional_association/KRTCAP2.pdf]

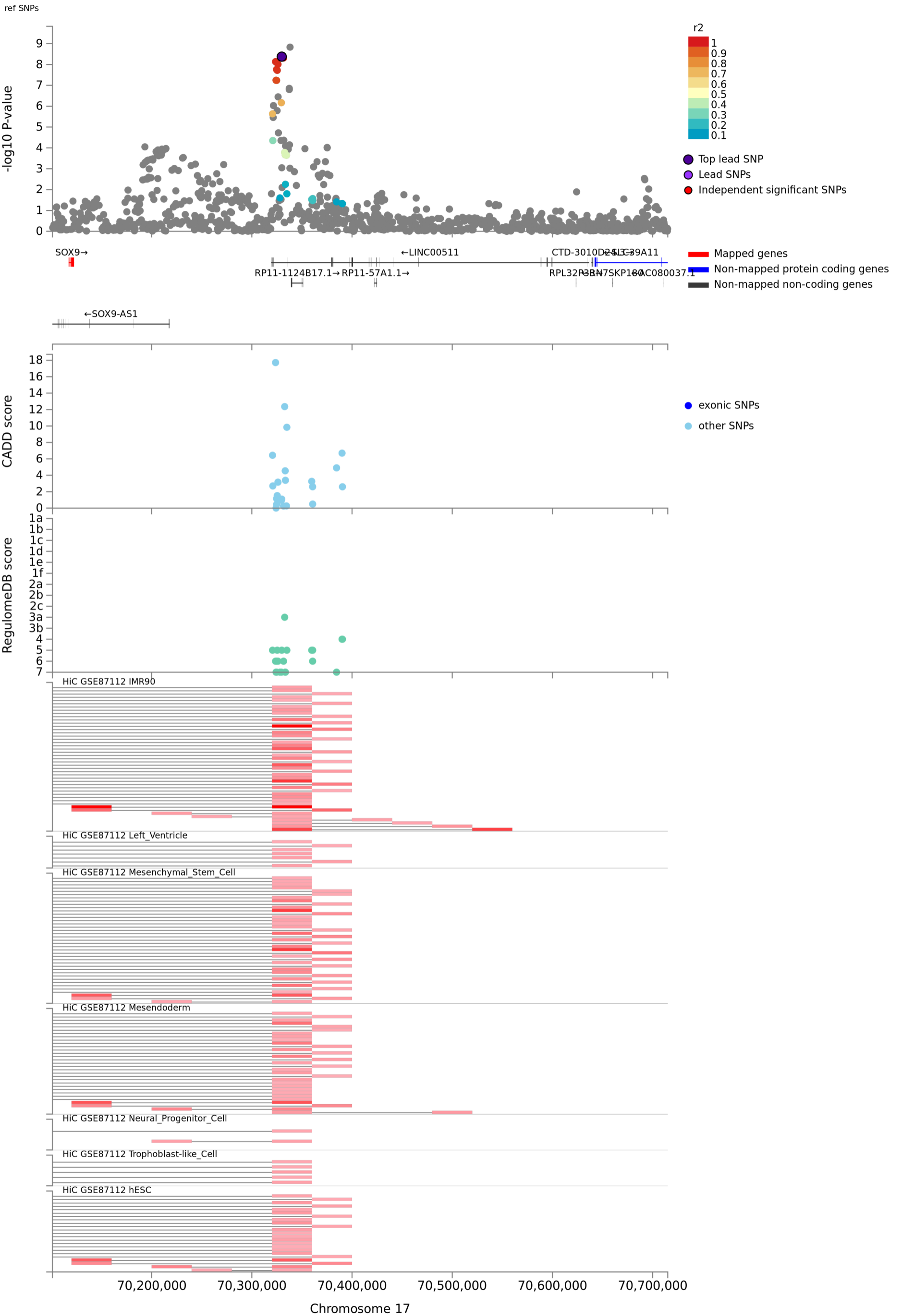

No eQTL of selected tissues exists in this region.

Supplement: Supplementary file 8 — Supplementary Dataset 5 [file 41467_2019_10630_MOESM8_ESM.zip › regional_association/LOC100499467.pdf]

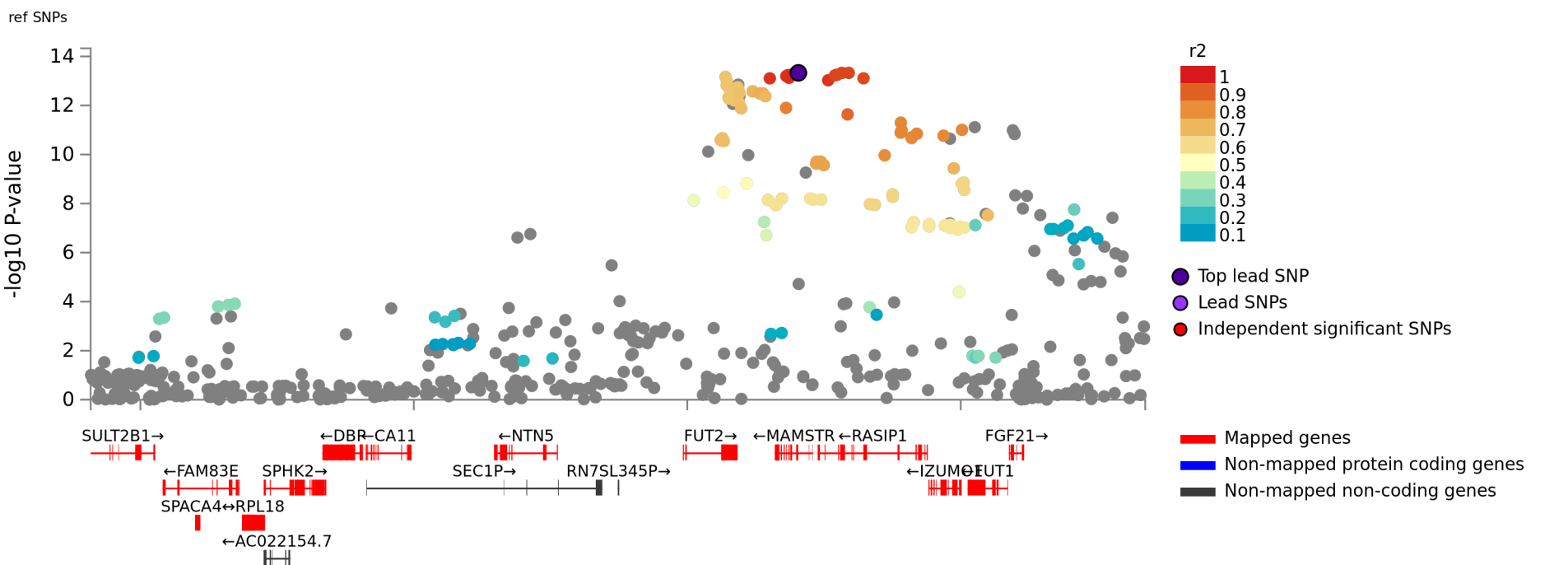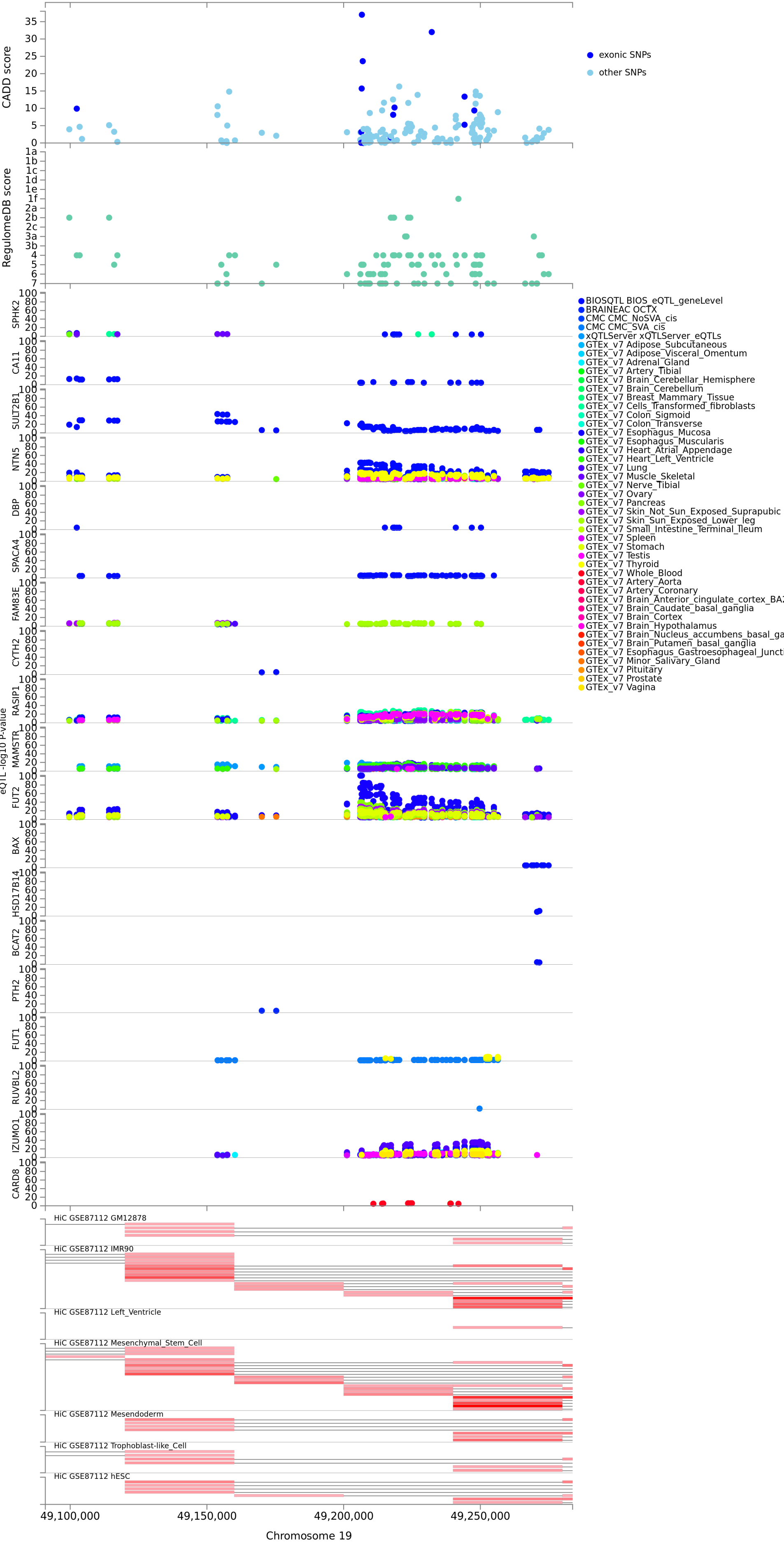

Supplement: Supplementary file 8 — Supplementary Dataset 5 [file 41467_2019_10630_MOESM8_ESM.zip › regional_association/MAMSTR.pdf]

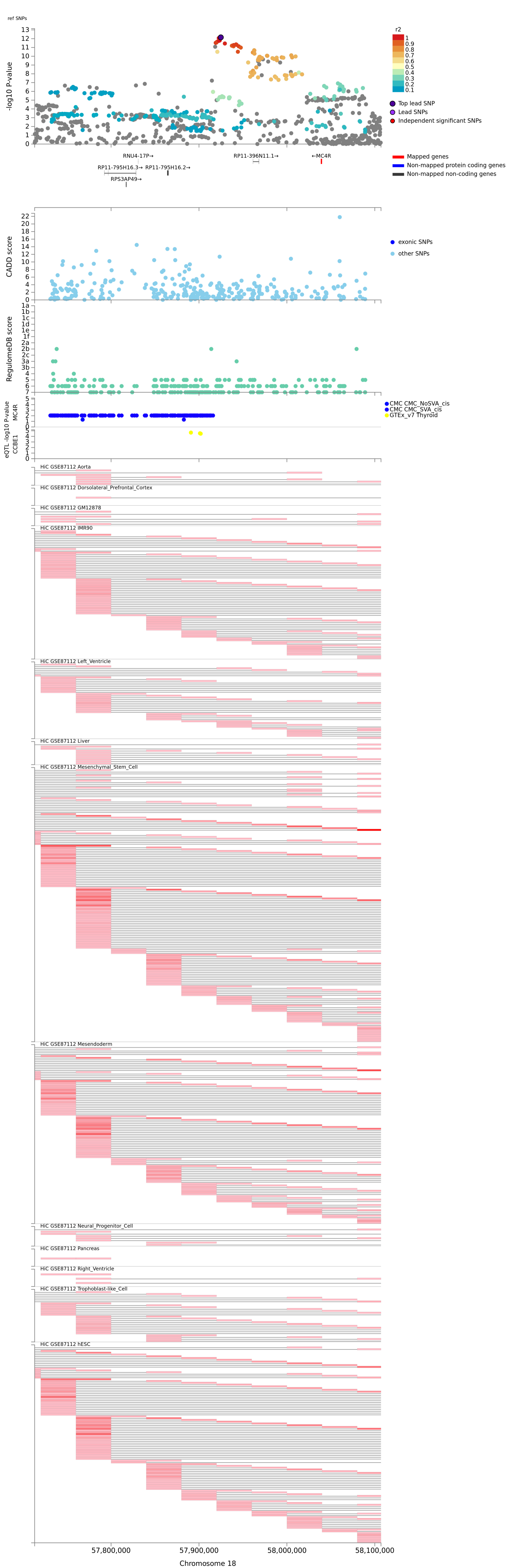

Supplement: Supplementary file 8 — Supplementary Dataset 5 [file 41467_2019_10630_MOESM8_ESM.zip › regional_association/MC4R.pdf]

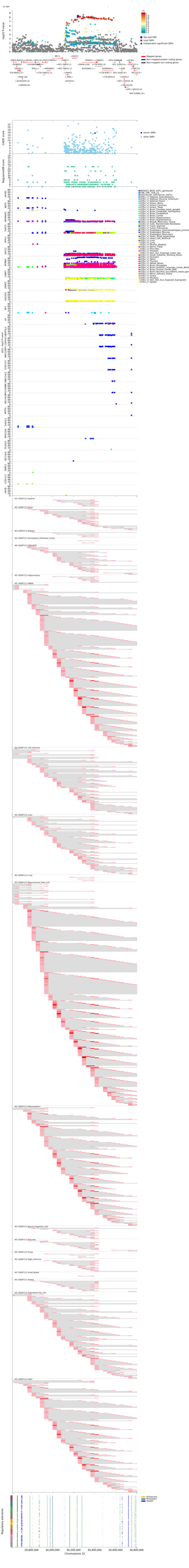

Supplement: Supplementary file 8 — Supplementary Dataset 5 [file 41467_2019_10630_MOESM8_ESM.zip › regional_association/MTMR_4.pdf]

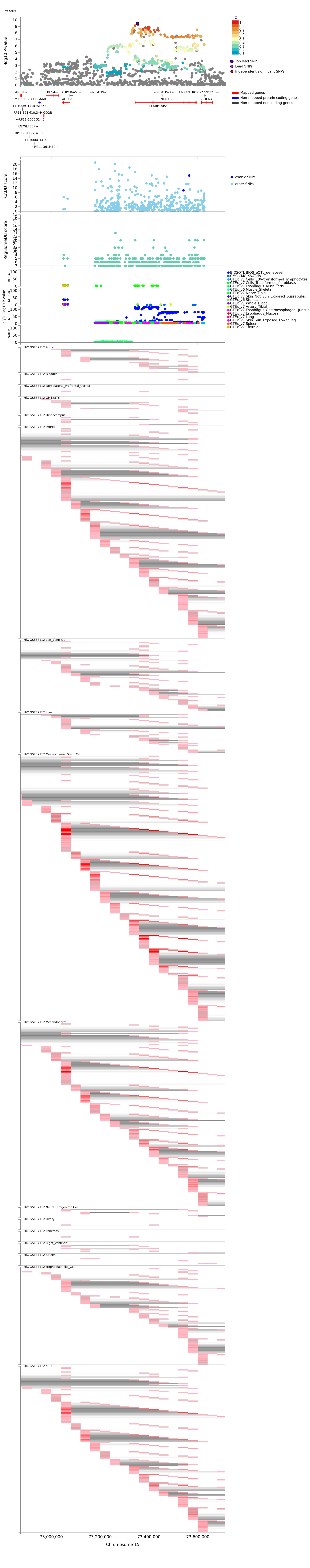

Supplement: Supplementary file 8 — Supplementary Dataset 5 [file 41467_2019_10630_MOESM8_ESM.zip › regional_association/NEO1.pdf]

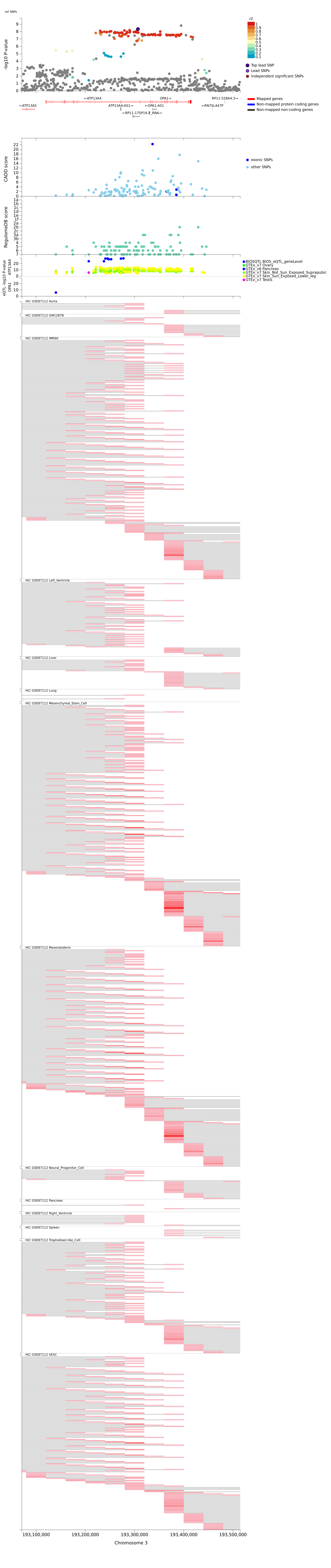

Supplement: Supplementary file 8 — Supplementary Dataset 5 [file 41467_2019_10630_MOESM8_ESM.zip › regional_association/OPA1.pdf]

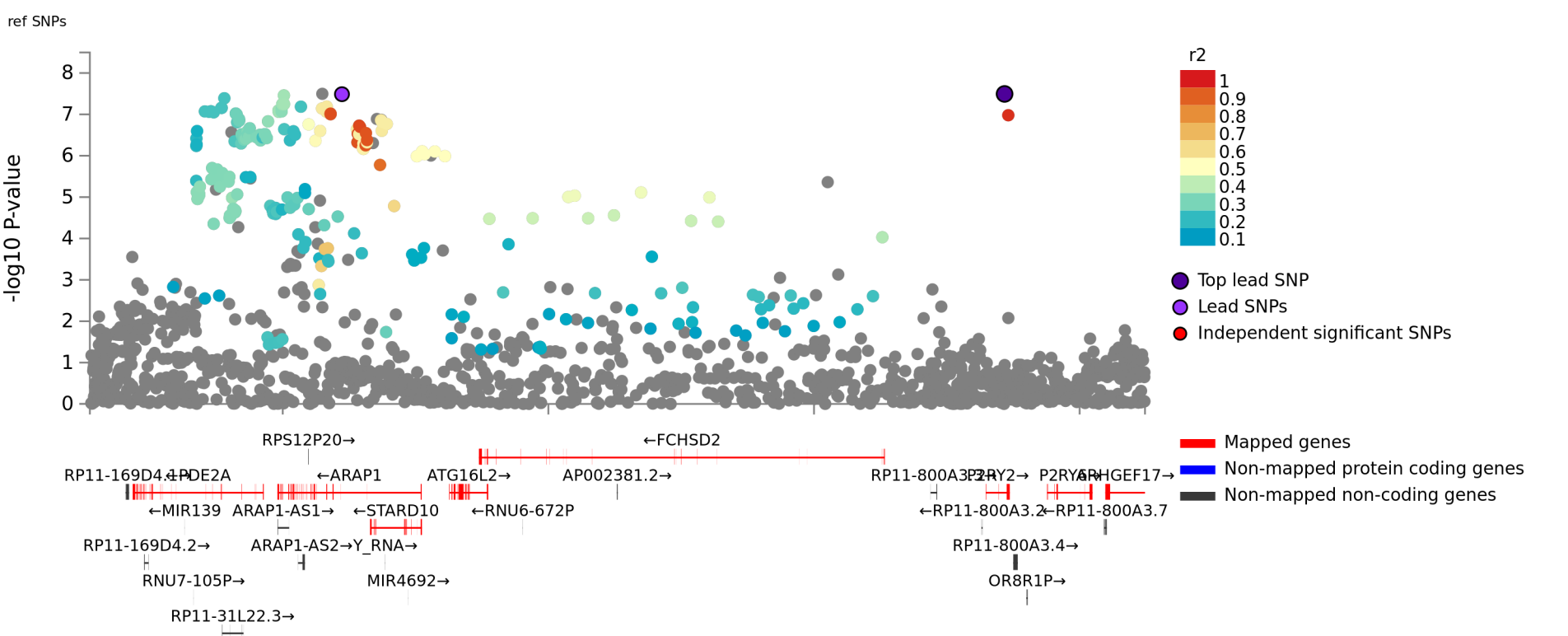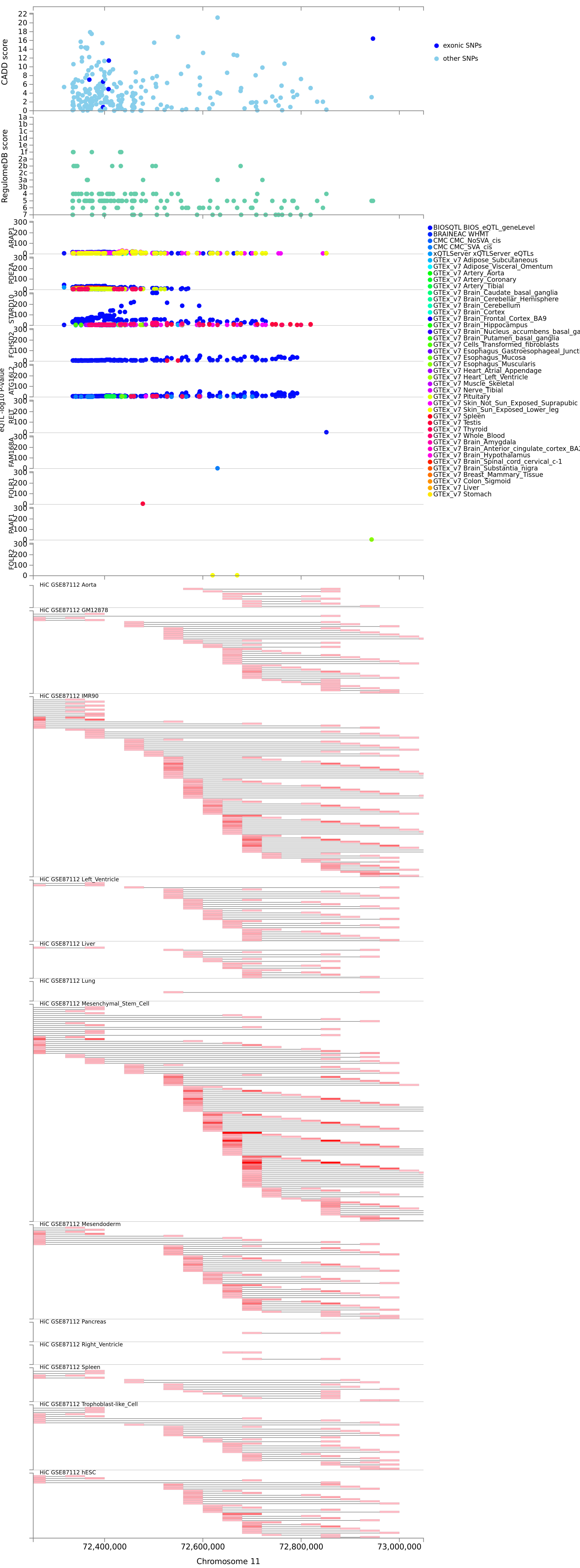

Supplement: Supplementary file 8 — Supplementary Dataset 5 [file 41467_2019_10630_MOESM8_ESM.zip › regional_association/P2RY2.pdf]

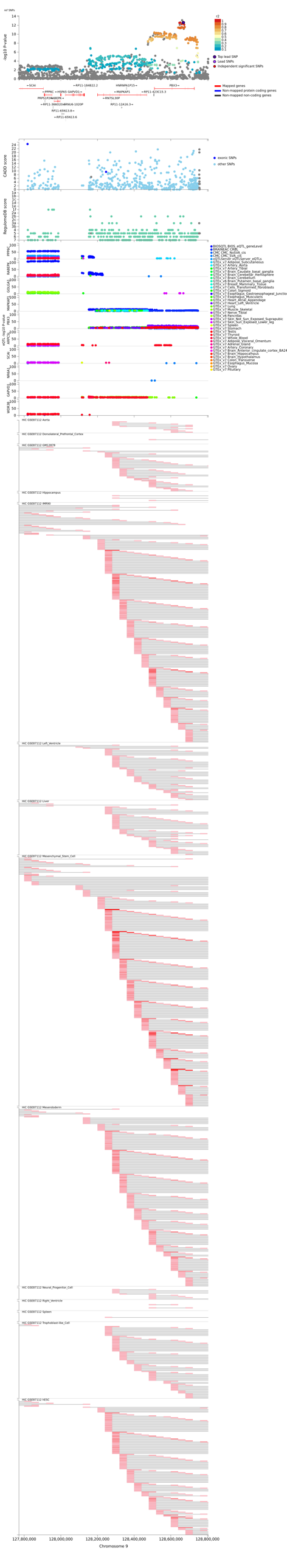

Supplement: Supplementary file 8 — Supplementary Dataset 5 [file 41467_2019_10630_MOESM8_ESM.zip › regional_association/PBX3.pdf]

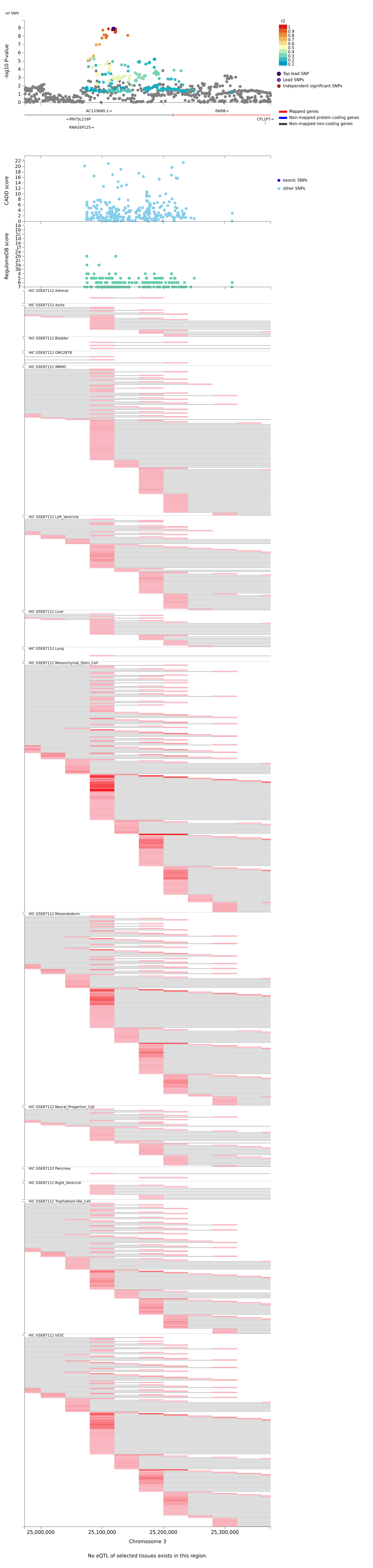

Supplement: Supplementary file 8 — Supplementary Dataset 5 [file 41467_2019_10630_MOESM8_ESM.zip › regional_association/RARB.pdf]

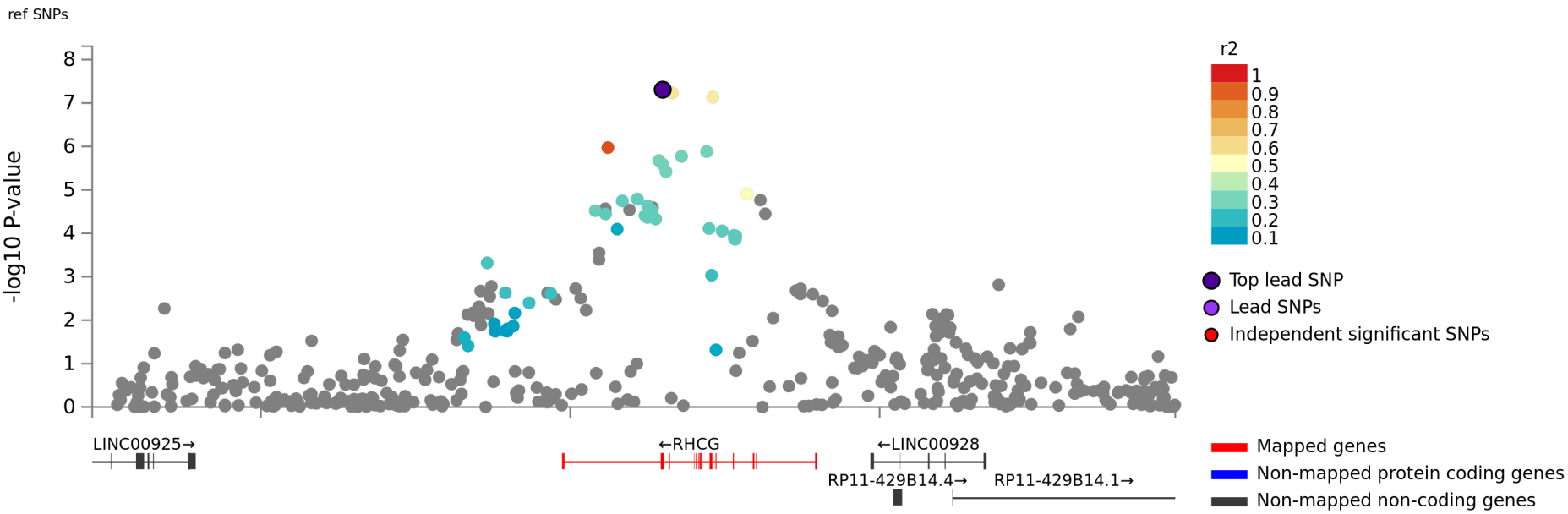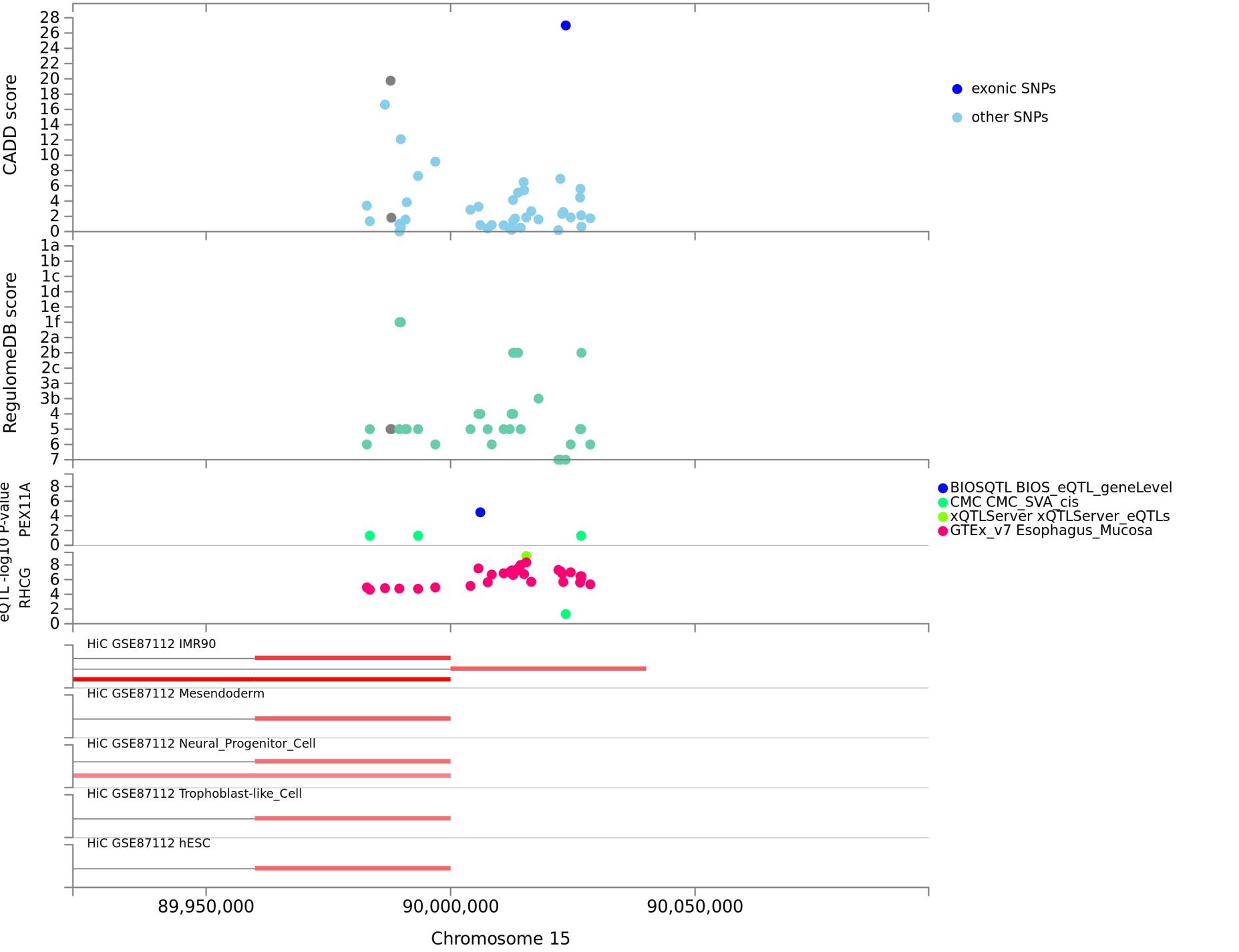

Supplement: Supplementary file 8 — Supplementary Dataset 5 [file 41467_2019_10630_MOESM8_ESM.zip › regional_association/RHCG.pdf]

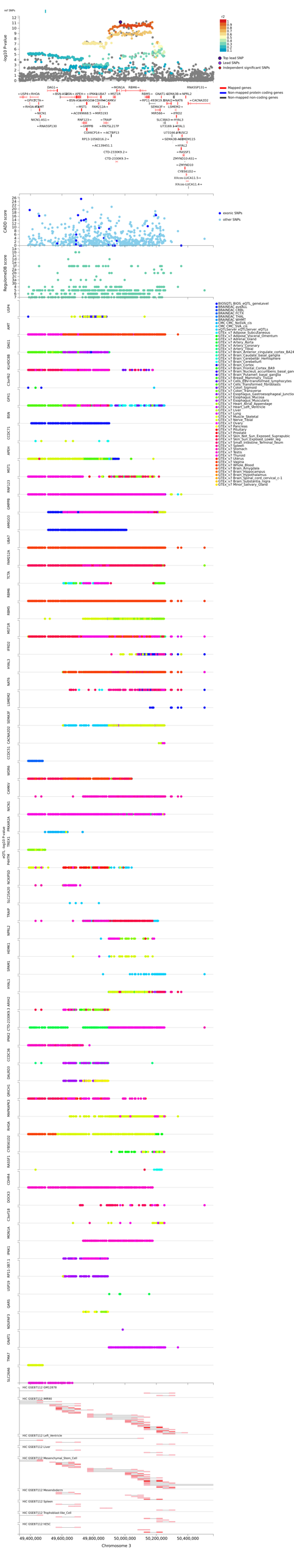

Supplement: Supplementary file 8 — Supplementary Dataset 5 [file 41467_2019_10630_MOESM8_ESM.zip › regional_association/RMB5.pdf]

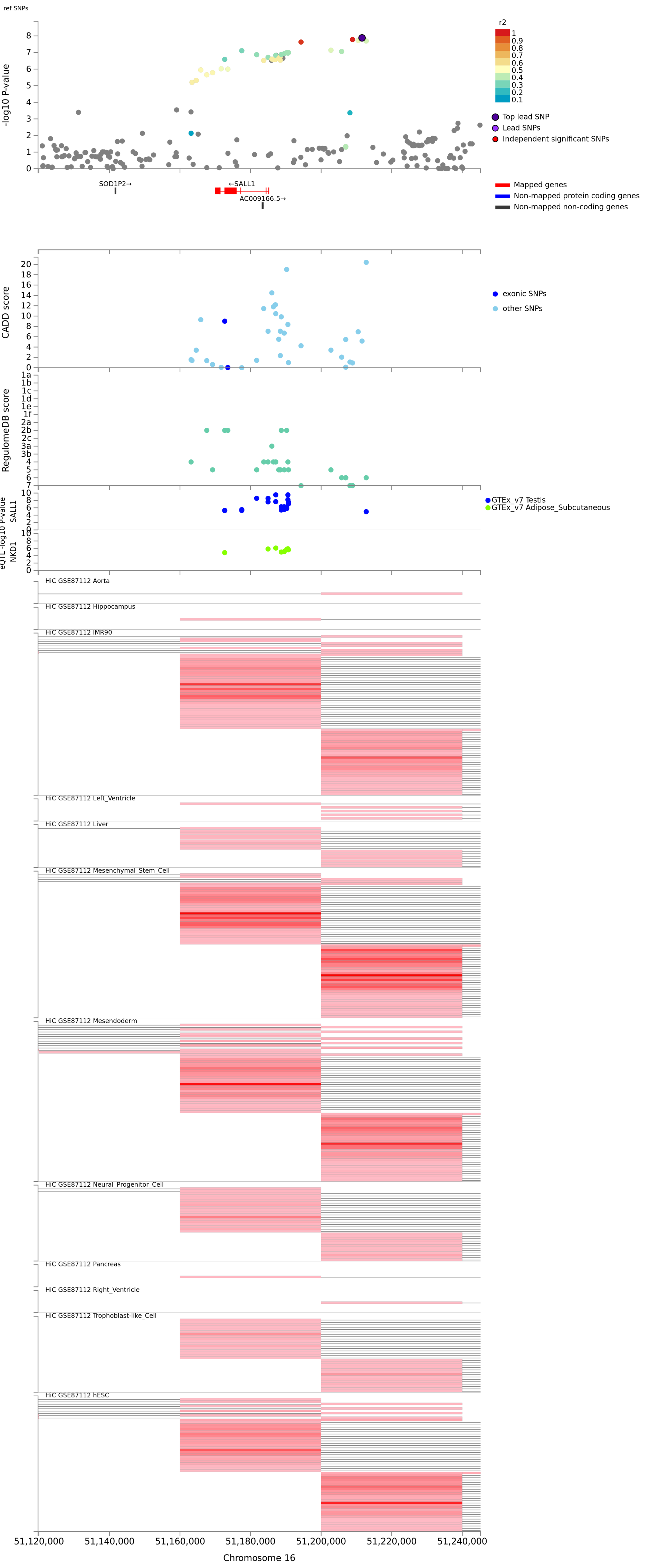

Supplement: Supplementary file 8 — Supplementary Dataset 5 [file 41467_2019_10630_MOESM8_ESM.zip › regional_association/SALL1.pdf]

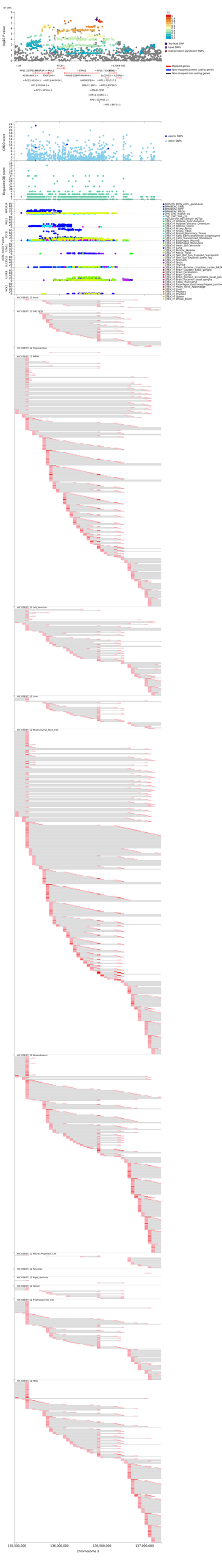

Supplement: Supplementary file 8 — Supplementary Dataset 5 [file 41467_2019_10630_MOESM8_ESM.zip › regional_association/STAG1.pdf]

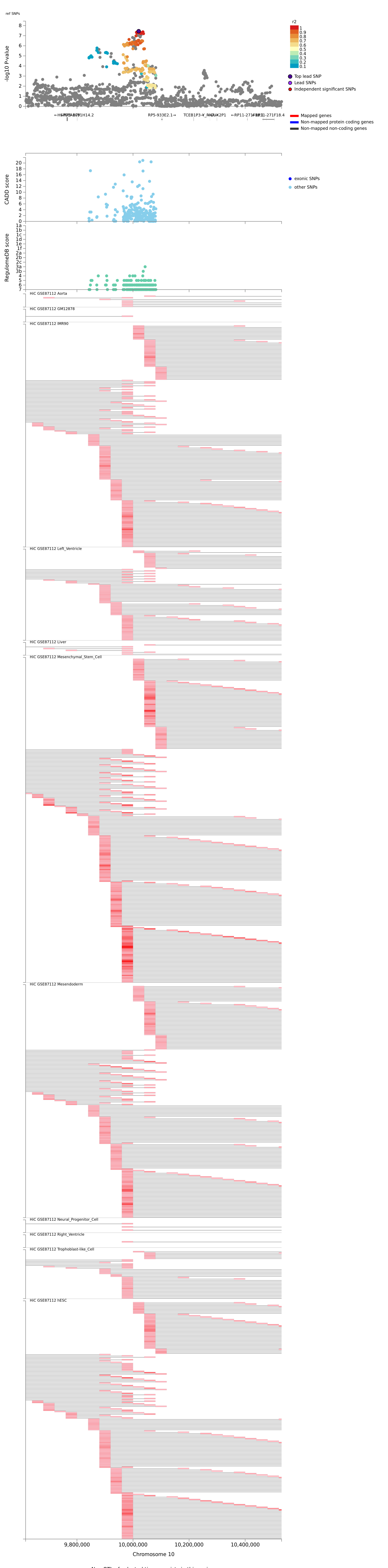

Supplement: Supplementary file 8 — Supplementary Dataset 5 [file 41467_2019_10630_MOESM8_ESM.zip › regional_association/STFA1P.pdf]

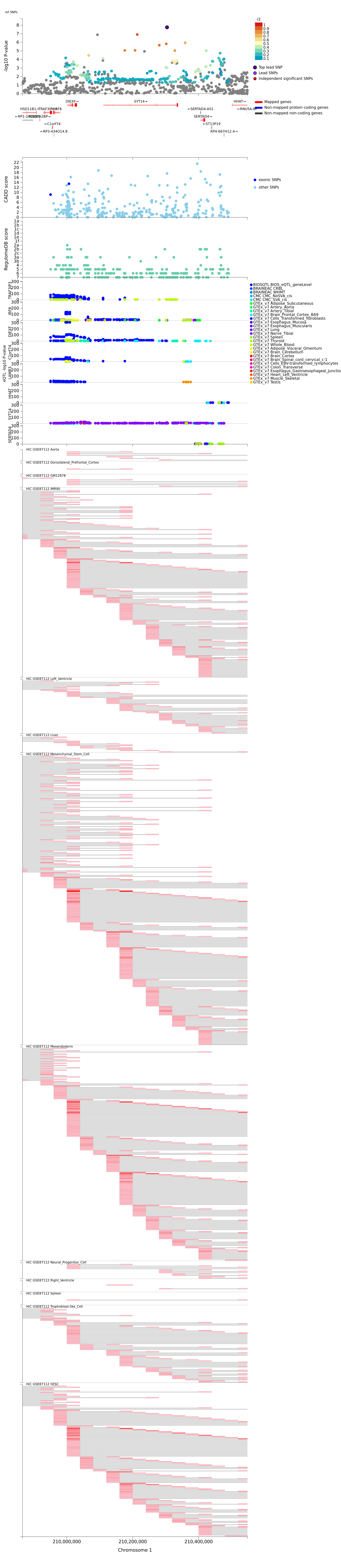

Supplement: Supplementary file 8 — Supplementary Dataset 5 [file 41467_2019_10630_MOESM8_ESM.zip › regional_association/SYT14.pdf]

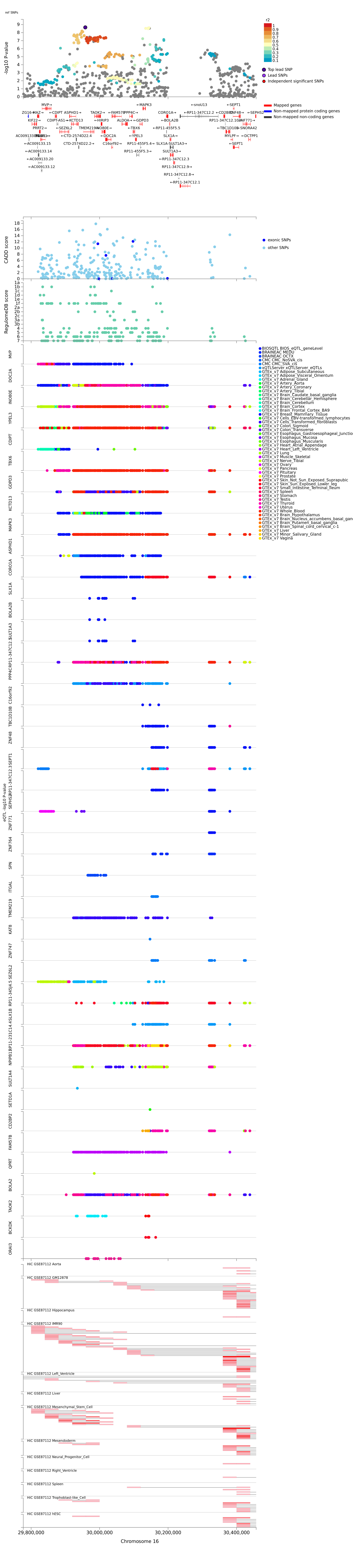

Supplement: Supplementary file 8 — Supplementary Dataset 5 [file 41467_2019_10630_MOESM8_ESM.zip › regional_association/TMEM219.pdf]

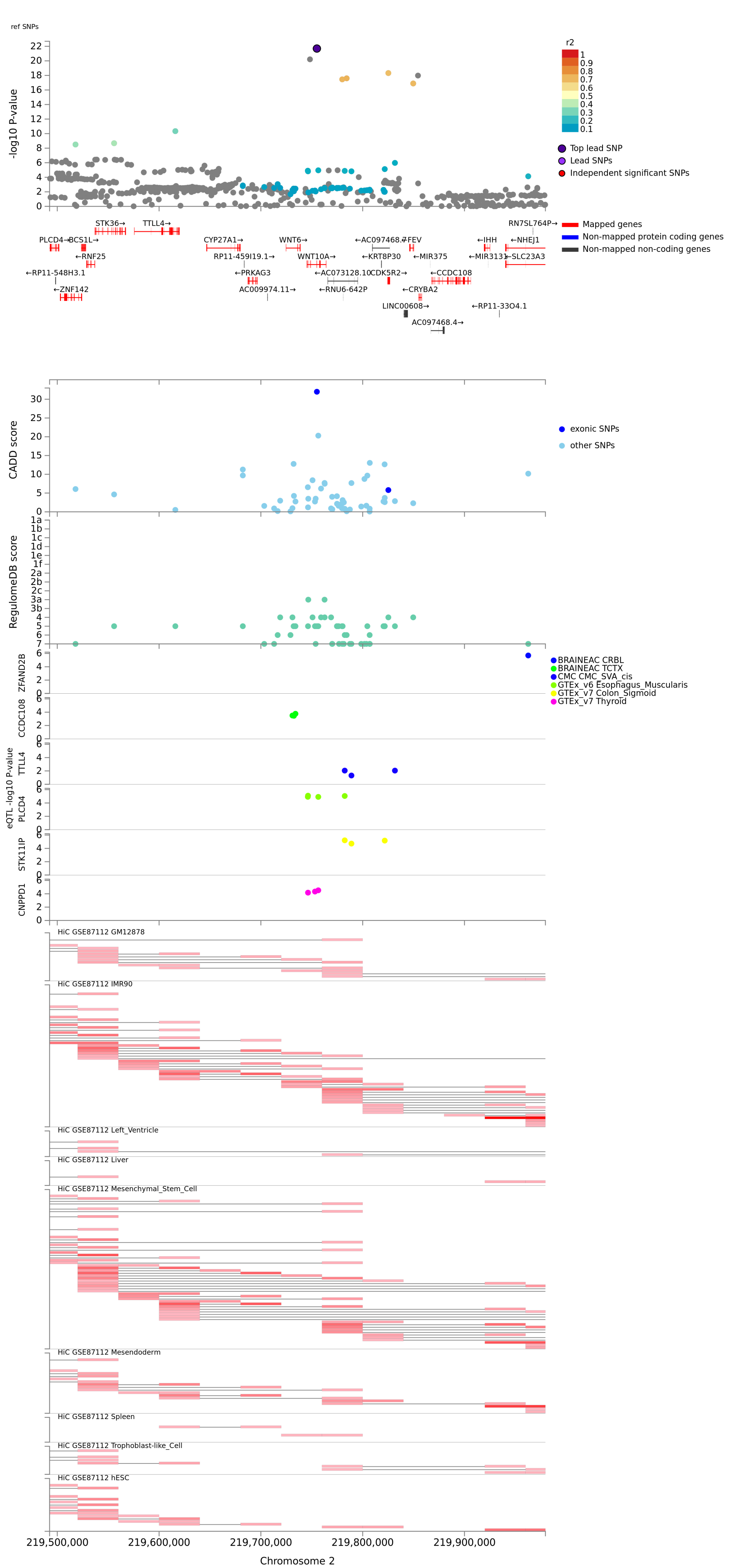

Supplement: Supplementary file 8 — Supplementary Dataset 5 [file 41467_2019_10630_MOESM8_ESM.zip › regional_association/WNT10A.pdf]

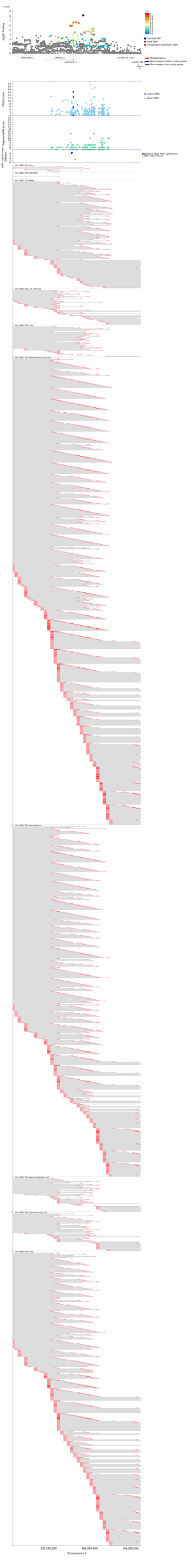

Supplement: Supplementary file 8 — Supplementary Dataset 5 [file 41467_2019_10630_MOESM8_ESM.zip › regional_association/ZNF804A.pdf]
