# Supplementary material for: Genome-wide analysis of dental caries and periodontitis combining clinical and self-reported data
Source: Nat Commun. 2019 Jun 24;10:2773. doi: 10.1038/s41467-019-10630-1 (PMC6591304; doi:10.1038/s41467-019-10630-1)
Supplement: Supplementary file 8 — Supplementary Dataset 5 [file FAM118A.pdf › regional_association/FAM118A.pdf]

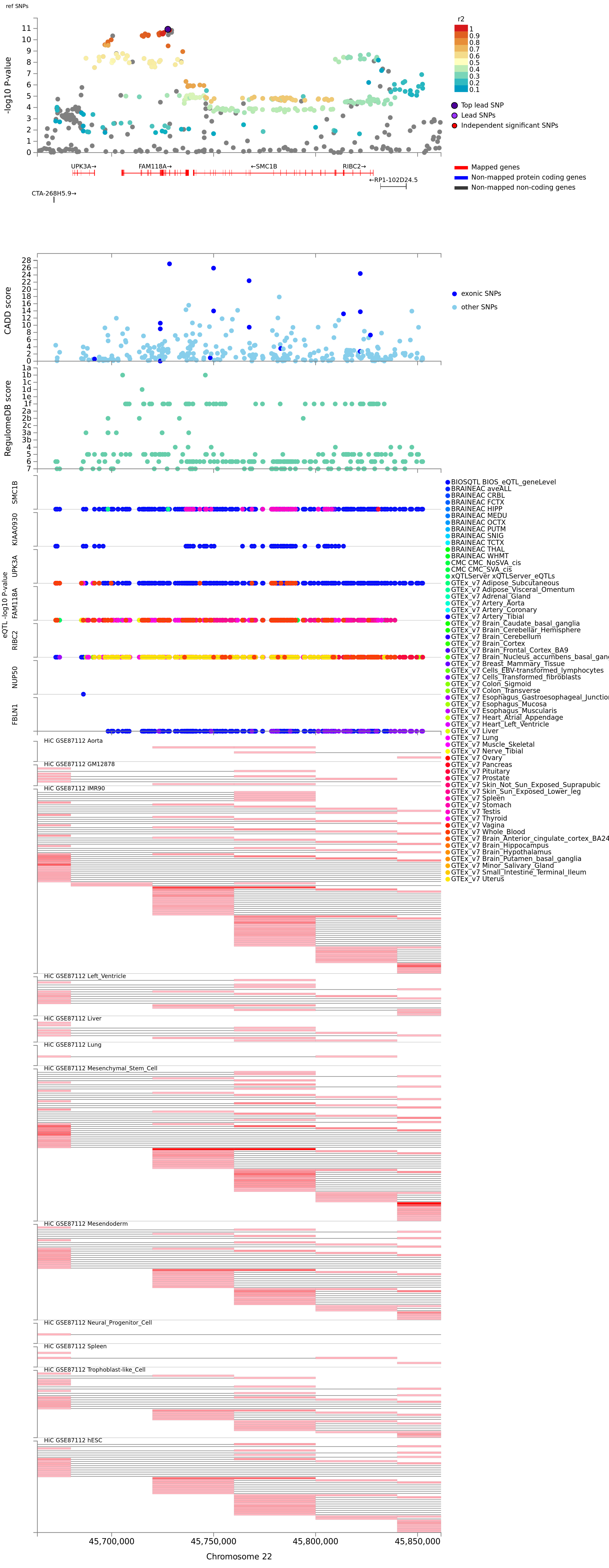

ref SNPs
-log10 P-value
r2
1
0.9
0.8
0.7
0.6
0.5
0.4
0.3
0.2
0.1
Top lead SNP
Lead SNPs
Independent significant SNPs
UPK3A→
FAM118A→
←SMC1B
RIBC2→
←RP1-102D24.5
CTA-268H5.9→
Mapped genes
Non-mapped protein coding genes
Non-mapped non-coding genes
CADD score
exonic SNPs
other SNPs
RegulomeDB score
eQTL -log10 P-value
SMC1B
KIAA0930
UPK3A
FAM118A
RIBC2
NUP50
FBLN1
BIOSQTL BIOS_eQTL_geneLevel
BRAINEAC aveALL
BRAINEAC CRBL
BRAINEAC FCTX
BRAINEAC HIPP
BRAINEAC MEDU
BRAINEAC OCTX
BRAINEAC PUTM
BRAINEAC SNIG
BRAINEAC TCTX
BRAINEAC THAL
BRAINEAC WHMT
CMC CMC_NoSVA_cis
CMC CMC_SVA_cis
xQTLServer xQTLServer_eQTLs
GTEx_v7 Adipose_Subcutaneous
GTEx_v7 Adipose_Visceral_Omentum
GTEx_v7 Adrenal_Gland
GTEx_v7 Artery_Aorta
GTEx_v7 Artery_Coronary
GTEx_v7 Artery_Tibial
GTEx_v7 Brain_Caudate_basal_ganglia
GTEx_v7 Brain_Cerebellar_Hemisphere
GTEx_v7 Brain_Cerebellum
GTEx_v7 Brain_Cortex
GTEx_v7 Brain_Frontal_Cortex_BA9
GTEx_v7 Brain_Nucleus_accumbens_basal_gang
GTEx_v7 Breast_Mammary_Tissue
GTEx_v7 Cells_EBV-transformed_lymphocytes
GTEx_v7 Cells_Transformed_fibroblasts
GTEx_v7 Colon_Sigmoid
GTEx_v7 Colon_Transverse
GTEx_v7 Esophagus_Gastroesophageal_Junction
GTEx_v7 Esophagus_Mucosa
GTEx_v7 Esophagus_Muscularis
GTEx_v7 Heart_Atrial_Appendage
GTEx_v7 Heart_Left_Ventricle
GTEx_v7 Liver
GTEx_v7 Lung
GTEx_v7 Muscle_Skeletal
GTEx_v7 Nerve_Tibial
GTEx_v7 Ovary
GTEx_v7 Pancreas
GTEx_v7 Pituitary
GTEx_v7 Prostate
GTEx_v7 Skin_Not_Sun_Exposed_Suprapubic
GTEx_v7 Skin_Sun_Exposed_Lower_leg
GTEx_v7 Spleen
GTEx_v7 Stomach
GTEx_v7 Testis
GTEx_v7 Thyroid
GTEx_v7 Vagina
GTEx_v7 Whole_Blood
GTEx_v7 Brain_Anterior_cingulate_cortex_BA24
GTEx_v7 Brain_Hippocampus
GTEx_v7 Brain_Hypothalamus
GTEx_v7 Brain_Putamen_basal_ganglia
GTEx_v7 Minor_Salivary_Gland
GTEx_v7 Small_Intestine_Terminal_Ileum
GTEx_v7 Uterus
HiC GSE87112 Aorta
HiC GSE87112 GM12878
HiC GSE87112 IMR90
HiC GSE87112 Left_Ventricle
HiC GSE87112 Liver
HiC GSE87112 Lung
HiC GSE87112 Mesenchymal_Stem_Cell
HiC GSE87112 Mesendoderm
HiC GSE87112 Neural_Progenitor_Cell
HiC GSE87112 Spleen
HiC GSE87112 Trophoblast-like_Cell
HiC GSE87112 hESC
45,700,000
45,750,000
45,800,000
45,850,000
Chromosome 22
