# Supplementary material for: Genome-wide analysis of dental caries and periodontitis combining clinical and self-reported data
Source: Nat Commun. 2019 Jun 24;10:2773. doi: 10.1038/s41467-019-10630-1 (PMC6591304; doi:10.1038/s41467-019-10630-1)
Supplement: Supplementary file 8 — Supplementary Dataset 5 [file PRUNE2.pdf › regional_association/PRUNE2.pdf]

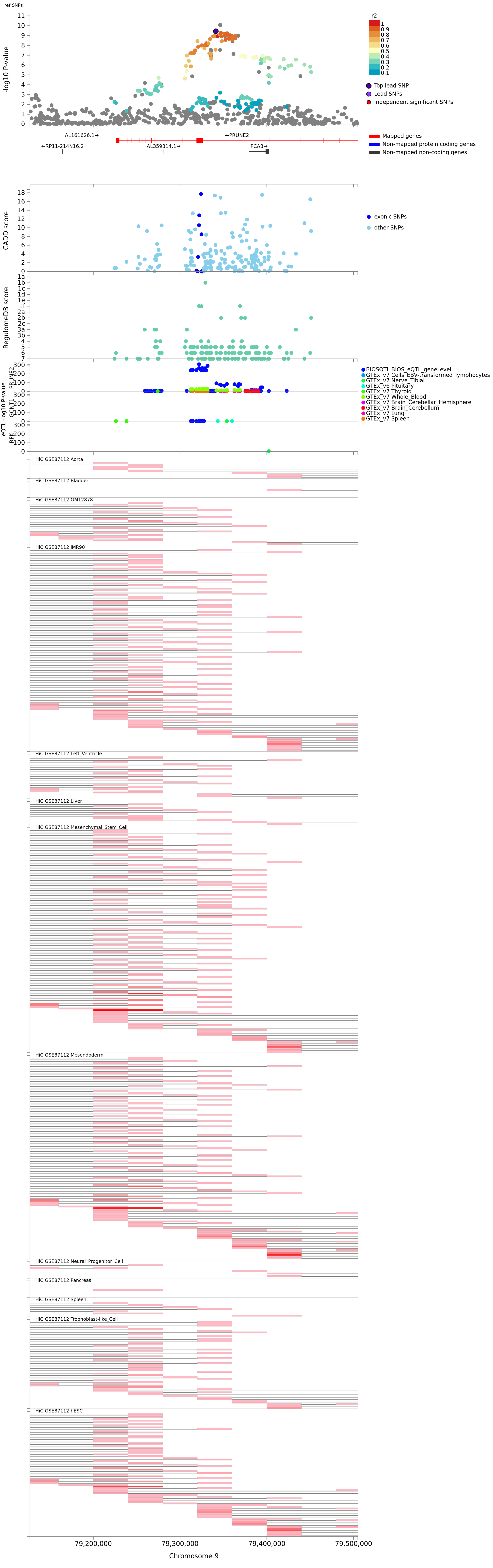
ref SNPs
-log10 P-value
r2
1
0.9
0.8
0.7
0.6
0.5
0.4
0.3
0.2
0.1
Top lead SNP
Lead SNPs
Independent significant SNPs
AL161626.1→
←PRUNE2
←RP11-214N16.2
AL359314.1→
PCA3→
Mapped genes
Non-mapped protein coding genes
Non-mapped non-coding genes
CADD score
exonic SNPs
other SNPs
RegulomeDB score
eQTL -log10 P-value
PRUNE2
GCNT1
RFK
BIOSQTL BIOS_eQTL_geneLevel
GTEx_v7 Cells_EBV-transformed_lymphocytes
GTEx_v7 Nerve_Tibial
GTEx_v6 Pituitary
GTEx_v7 Thyroid
GTEx_v7 Whole_Blood
GTEx_v7 Brain_Cerebellar_Hemisphere
GTEx_v7 Brain_Cerebellum
GTEx_v7 Lung
GTEx_v7 Spleen
HiC GSE87112 Aorta
HiC GSE87112 Bladder
HiC GSE87112 GM12878
HiC GSE87112 IMR90
HiC GSE87112 Left_Ventricle
HiC GSE87112 Liver
HiC GSE87112 Mesenchymal_Stem_Cell
HiC GSE87112 Mesendoderm
HiC GSE87112 Neural_Progenitor_Cell
HiC GSE87112 Pancreas
HiC GSE87112 Spleen
HiC GSE87112 Trophoblast-like_Cell
HiC GSE87112 hESC
79,200,000
79,300,000
79,400,000
79,500,000
Chromosome 9
