# Supplementary material for: Genome-wide analysis of dental caries and periodontitis combining clinical and self-reported data
Source: Nat Commun. 2019 Jun 24;10:2773. doi: 10.1038/s41467-019-10630-1 (PMC6591304; doi:10.1038/s41467-019-10630-1)
Supplement: Supplementary file 19 — Reporting Summary [file 41467_2019_10630_MOESM19_ESM.pdf]

nature research

Corresponding author(s): S. Haworth

Last updated by author(s): May 17, 2019

Reporting Summary

Nature Research wishes to improve the reproducibility of the work that we publish. This form provides structure for consistency and transparency in reporting. For further information on Nature Research policies, see Authors & Referees and the Editorial Policy Checklist.

Statistics

For all statistical analyses, confirm that the following items are present in the figure legend, table legend, main text, or Methods section.

n/a	Confirmed	
☐	☒	The exact sample size (*n*) for each experimental group/condition, given as a discrete number and unit of measurement
☐	☒	A statement on whether measurements were taken from distinct samples or whether the same sample was measured repeatedly
☐	☒	The statistical test(s) used AND whether they are one- or two-sided<br>*Only common tests should be described solely by name; describe more complex techniques in the Methods section.*
☐	☒	A description of all covariates tested
☐	☒	A description of any assumptions or corrections, such as tests of normality and adjustment for multiple comparisons
☐	☒	A full description of the statistical parameters including central tendency (e.g. means) or other basic estimates (e.g. regression coefficient) AND variation (e.g. standard deviation) or associated estimates of uncertainty (e.g. confidence intervals)
☐	☒	For null hypothesis testing, the test statistic (e.g. *F*, *t*, *r*) with confidence intervals, effect sizes, degrees of freedom and *P* value noted<br>*Give P values as exact values whenever suitable.*
☒	☐	For Bayesian analysis, information on the choice of priors and Markov chain Monte Carlo settings
☒	☐	For hierarchical and complex designs, identification of the appropriate level for tests and full reporting of outcomes
☒	☐	Estimates of effect sizes (e.g. Cohen's *d*, Pearson's *r*), indicating how they were calculated

Our web collection on statistics for biologists contains articles on many of the points above.

Software and code

Policy information about availability of computer code

Data collection	No new data were collected for this software
Data analysis	Analysis used a range of publicly available software tools. The names and (where appropriate) versions numbers and URLs are provided in the Methods.

For manuscripts utilizing custom algorithms or software that are central to the research but not yet described in published literature, software must be made available to editors/reviewers. We strongly encourage code deposition in a community repository (e.g. GitHub). See the Nature Research guidelines for submitting code & software for further information.

Data

Policy information about availability of data

All manuscripts must include a data availability statement. This statement should provide the following information, where applicable:

- Accession codes, unique identifiers, or web links for publicly available datasets
- A list of figures that have associated raw data
- A description of any restrictions on data availability

Genome-wide summary statistics of the analyses have been deposited in an online repository and are available at (URL: https://data.bris.ac.uk/data/dataset/2j2rqgzedxlq02oqbb4vmycnc2)
Genome-wide summary statistics for all metabolic and cardiovascular outcomes used in this analysis were downloaded from the sources listed in Supplementary Note 1.
Genome-wide summary statistics are available for the COHRA and DRDR projects' dental caries GWAS through the Human Genomics Analysis Interface of the FaceBase consortium (URL: http://FaceBase.sdmgenetics.pitt.edu/, NIH Grant # 5U01-DE024425).
Participant-level genomic and phenotypic data for the COHRA and DRDR projects are available through dbGaP (URL: https://www.ncbi.nlm.nih.gov/gap/; dbGaP Study Accession #: phs000095.v3.p1). Access to UKBiobank data is through a manged open access procedure which is described in full online (URL: http://www.ukbiobank.ac.uk/using-the-resource/). Source data for Fig 1.a are provided in Supplementary Table 17 and source data for Fig. 1b are provided in

Supplementary Table 18. Source data for Figures 3 and 4 are provided in Supplementary Tables 9 and 10.

Field-specific reporting

Please select the one below that is the best fit for your research. If you are not sure, read the appropriate sections before making your selection.

☒ Life sciences ☐ Behavioural & social sciences ☐ Ecological, evolutionary & environmental sciences

For a reference copy of the document with all sections, see nature.com/documents/nr-reporting-summary-flat.pdf

Life sciences study design

All studies must disclose on these points even when the disclosure is negative.

Sample size	No new data collection was performed so no sample size calculations are included
Data exclusions	Participants were excluded from analysis for a) missing phenotype data and b) genetic quality control measures. These are described in full in the Methods and Supplementary Data 1 and 2.
Replication	A single-sample design was undertaken with no formal replication stage. However, consistency in genetic effect in independent parts of the combined sample was assessed by comparing concordance in effect size and direction between GLIDE and UK Biobank (Figure 2 b and Figure 2 d).
Randomization	No intervention was performed so participants were not randomized
Blinding	No intervention was performed so participants or examiners were not blinded.

Reporting for specific materials, systems and methods

We require information from authors about some types of materials, experimental systems and methods used in many studies. Here, indicate whether each material, system or method listed is relevant to your study. If you are not sure if a list item applies to your research, read the appropriate section before selecting a response.

Materials & experimental systems

n/a	Involved in the study	
☒	☐	Antibodies
☒	☐	Eukaryotic cell lines
☒	☐	Palaeontology
☒	☐	Animals and other organisms
☐	☒	Human research participants
☒	☐	Clinical data

Methods

n/a	Involved in the study	
☒	☐	ChIP-seq
☒	☐	Flow cytometry
☒	☐	MRI-based neuroimaging

Human research participants

Policy information about studies involving human research participants

Population characteristics	All studies included adults (aged 18 years and older) who had consented to participate in research. Analysis included participants of European ancestry, admixed Hispanic/Latino ancestry and East Asian ancestry as described in Supplementary Data 1
Recruitment	The participating cohorts used a range of recruitment strategies as described in Supplementary Data 1.
Ethics oversight	Numerous ethnics committees provided approval for the participating studies. These are listed in Supplementary Data 1.

Note that full information on the approval of the study protocol must also be provided in the manuscript.
